# Evidence for Innate and Adaptive Immune Responses in a Cohort of Intractable Pediatric Epilepsy Surgery Patients

**DOI:** 10.3389/fimmu.2019.00121

**Published:** 2019-01-29

**Authors:** Geoffrey C. Owens, Alejandro J. Garcia, Aaron Y. Mochizuki, Julia W. Chang, Samuel D. Reyes, Noriko Salamon, Robert M. Prins, Gary W. Mathern, Aria Fallah

**Affiliations:** ^1^Department of Neurosurgery, David Geffen School of Medicine, University of California, Los Angeles, Los Angeles, CA, United States; ^2^Division of Hematology Oncology, Department of Medicine, David Geffen School of Medicine, University of California, Los Angeles, Los Angeles, CA, United States; ^3^Department of Pediatrics, David Geffen School of Medicine, University of California, Los Angeles, Los Angeles, CA, United States; ^4^Intellectual and Developmental Disabilities Research Center, David Geffen School of Medicine, University of California, Los Angeles, Los Angeles, CA, United States; ^5^Department of Radiological Sciences, David Geffen School of Medicine at the University of California, Los Angeles, Los Angeles, CA, United States; ^6^Department of Molecular and Medical Pharmacology, David Geffen School of Medicine, University of California, Los Angeles, Los Angeles, CA, United States; ^7^Brain Research Institute, David Geffen School of Medicine, University of California, Los Angeles, Los Angeles, CA, United States; ^8^Parker Institute for Cancer Immunotherapy, David Geffen School of Medicine, University of California, Los Angeles, Los Angeles, CA, United States; ^9^Mattel Children's Hospital, David Geffen School of Medicine, University of California, Los Angeles, Los Angeles, CA, United States

**Keywords:** brain, epilepsy, inflammation, peripheral immune cells, mass cytometry (CyTOF)

## Abstract

Brain-infiltrating lymphocytes (BILs) were isolated from resected brain tissue from 10 pediatric epilepsy patients who had undergone surgery for Hemimegalencephaly (HME) (*n* = 1), Tuberous sclerosis complex (TSC) (*n* = 2), Focal cortical dysplasia (FCD) (*n* = 4), and Rasmussen encephalitis (RE) (*n* = 3). Peripheral blood mononuclear cells (PBMCs) were also isolated from blood collected at the time of the surgery. Cells were immunostained with a panel of 20 antibody markers, and analyzed by mass cytometry. To identify and quantify the immune cell types in the samples, an unbiased clustering method was applied to the entire data set. More than 85 percent of the CD45^+^ cells isolated from resected RE brain tissue comprised T cells; by contrast NK cells and myeloid cells constituted 80–95 percent of the CD45^+^ cells isolated from the TSC and the FCD brain specimens. Three populations of myeloid cells made up >50 percent of all of the myeloid cells in all of the samples of which a population of HLA-DR^+^ CD11b^+^ CD4^−^ cells comprised the vast majority of myeloid cells in the BIL fractions from the FCD and TSC cases. CD45RA^+^ HLA-DR^−^ CD11b^+^ CD16^+^ NK cells constituted the major population of NK cells in the blood from all of the cases. This subset also comprised the majority of NK cells in BILs from the resected RE and HME brain tissue, whereas NK cells defined as CD45RA^−^ HLA-DR^+^ CD11b^−^ CD16^−^ cells comprised 86–96 percent of the NK cells isolated from the FCD and TSC brain tissue. Thirteen different subsets of CD4 and CD8 αβ T cells and γδ T cells accounted for over 80% of the CD3^+^ T cells in all of the BIL and PBMC samples. At least 90 percent of the T cells in the RE BILs, 80 percent of the T cells in the HME BILs and 40–66 percent in the TSC and FCD BILs comprised activated antigen-experienced (CD45RO^+^ HLA-DR^+^ CD69^+^) T cells. We conclude that even in cases where there is no evidence for an infection or an immune disorder, activated peripheral immune cells may be present in epileptogenic areas of the brain, possibly in response to seizure-driven brain inflammation.

## Introduction

It has been estimated that by 15 years of age, approximately one percent of children will have experienced at least one seizure (1). For reasons that may not always be understood ~10% of children go on to develop medically refractory epilepsy, which is defined as a failure of two or more antiepileptic drugs to control seizures, and at least one seizure per month for ≥18 months (2). Recurrent seizures may severely impair a child's cognitive development leading to lifelong learning and behavioral difficulties. For children with drug-resistant epilepsy, surgery may be the only option to obtain seizure freedom, but will result in neurological deficits if the zone of resection involves eloquent cerebral cortex.

Many of the children who are candidates for epilepsy surgery suffer from rare neurological disorders including Rasmussen encephalitis (RE), Tuberous Sclerosis Complex (TSC), Focal Cortical Dysplasia (FCD), and Hemimegalencephaly (HME). RE patients present with partial (focal) seizures; magnetic resonance images (MRI) may indicate inflammation and atrophy in the affected cerebral hemisphere (3). The inflammation may spread through the affected cerebral hemisphere, but generally does not cross over to the contralateral hemisphere (4). Histopathological examination of resected brain tissue and brain biopsies show T cells in perivascular spaces, leptomeninges, and in small clusters scattered throughout the affected gray and white matter (5, 6). Clonally focused T cells have been found in resected RE brain tissue strongly implicating an antigen driven immune response in disease etiology (7–10).

In TSC patients, germ line and somatic dominant loss of function mutations in the genes encoding hamartin (TSC1) or tuberin (TSC2) can cause the development of benign tumors and, abnormally differentiated cortical neuronal progenitors that may cause focal seizures (11). Tuberin and harmatin are components of a complex that regulates the activity of the protein kinase, mTOR (12). Similarly, FCD and HME can be caused by activating somatic mutations in the MTOR gene, and in genes that regulate mTOR activity (13–15). Histopathological examination of resected brain tissue and analysis of RNA transcripts has shown that FCD and TSC lesions may be associated with an inflammatory reaction (11, 16–19).

In the present study we report on the characterization, by mass cytometry, of brain-infiltrating lymphocytes (BILs) isolated from surgical resections of epileptogenic tissue to treat FCD, TSC, and HME, as well as RE, and of peripheral blood mononuclear cells (PBMCs) prepared from blood collected at the time of surgery from the same cases. Immune cell profiling showed that activated T cells were present in brain tissue from all of the cases examined, and that the relative abundance of adaptive and innate lymphoid cells, and myeloid cells markedly differed between the RE and non-RE cases.

## Methods

### Patient Cohort

Under UCLA IRB approval (IRB#18-000154) brain tissue and blood were collected at surgery as part of UCLA's Pediatric Epilepsy Surgery Program. All of the patients or their parents or legal guardians provided informed consent for the use of the surgical remnant and blood for research purposes. All specimens were collected using the same standard operating procedures. De-identified patient information including age at seizure onset, age at surgery, gender, and affected cerebral hemisphere was collected with informed consent (Table S1).

### Isolation of Peripheral Blood Lymphocytes and Brain-Infiltrating Lymphocytes

PBMCs were isolated by density gradient centrifugation using Ficoll-Paque PLUS (GE Healthcare, Piscataway, NJ). BILs were isolated from collagenase-treated brain tissue by fractionation on a step gradient (20). Briefly, brain tissue was diced manually on ice in dissociation solution (HBSS with 20 mM HEPES pH 7.0, 5 mM glucose, and 50 U/ml penicillin/streptomycin). Tissue fragments were incubated with agitation at room temperature overnight in dissociation solution containing 0.5 mg/ml Type IV collagenase (Worthington Biochemical Corp., Lakewood, NJ) and 5% filtered human serum (Mediatech Inc., Manassas, VA). The dissociated tissue was fractionated on a 30%: 70% Percoll^®;^ (SigmaAldrich, St. Louis, MO) step gradient in RPMI containing 20 mM HEPES. PBMCs and BILs were cryopreserved in 90% human serum /10% DMSO.

### Multiparameter Mass Cytometry

The panel comprised the following markers: CD45 (89Y or 154Sm), CD196 (141Pr), CD19 (142Nd), CD69 (144Nd), CD4 (145Nd), CD8 (146Nd), CD25 (149Sm), CD103 (151Eu), CD45RA (155Gd), CD183 (156Gd), CD56 (163Dy), CD45RO (164Dy), CD16 (165Ho), TCRγδ (168Er), CD3 (170Er), CD195 (171Yb), HLA-DR (174Yb), CD194 (175Yb), CD127 (176Yb), and CD11b (209Bi). All metal-tagged antibodies (Abs) were obtained from Fluidigm (San Francisco, CA) except the CD8 and TCRγδ antibodies, which were conjugated in-house. PBMCs and BILs were stained according to Fluidigm's protocol. In brief, cells were thawed, and washed in phosphate-buffered saline (PBS); prior to staining BILs were filtered through a 40 μm sieve to remove any aggregates. Cells were resuspended in 1 ml of PBS and stained for 5 min with 1 μM Cisplatin. After quenching the staining with Maxpar^®^ cell staining buffer (Fluidigm), cells were incubated with the cocktail of Abs in 100 μl of Maxpar^®^ cell staining buffer for 30 min at room temperature. The Ab cocktail for staining PBMCs contained the CD45 Ab conjugated to Samarium 154 (154Sm) while the BIL Ab cocktail contained the CD45 Ab tagged with Yttrium 89 (89Y). Following two wash steps, cells were fixed overnight at 4°C in Maxpar^®^ fixation and permeabilization buffer containing 0.125 μM Intercalator-Ir (Fluidigm). BILs and PBMCs from the same surgery were combined at this point and washed twice with Maxpar^®^ cell staining buffer and a further two times with water. Having barcoded the BILs and PBMCs with a different metal-conjugated CD45 Ab it was possible to analyze them as single sample, thus the larger number of PBMCs served as a carrier for the smaller number of BILs that were isolated from brain tissue. Cells were resuspended in 10 percent EQ™ Four Element Calibration Beads (Fluidigm) containing Cerium (140/142Ce), Europium (151/153Eu), Holmium (165Ho), and Lutetium (175/176Lu). Samples were acquired on a Helios^®^ cytometry time of flight (CyTOF) system (Fluidigm) at an event rate of 300–500 events/s. Post-acquisition data normalization was done using bead-based normalization in the CyTOF software. Prior to analysis, data were gated to eliminate normalization beads, debris, dead cells, and doublets.

The analysis of FCS files was initially carried out using Cytobank (21). For each surgery case, the marker expression on the BILs was resolved by first gating live singlets on CD45 (89Y). Conversely marker expression on PBMCs was resolved by first gating on CD45 (154Sm). The data were then split into two separate FCS files using software tools in Cytobank (21). To define subsets of immune cells in each BIL and PBMC population, the entire high dimensional dataset (comprising 20 FCS files) was converted into a matrix of pair-wise similarities by implementing the t-based stochastic neighbor embedding (t-SNE) algorithm, followed by a density-based clustering method (ClusterX) (22). The resulting 2-D plots from this procedure were exported to CorelDraw2017 as portable document format files (Corel Corporation, Ottawa, Canada). Individual FCS files were analyzed using FlowJo^®^ software (FlowJo LLC, Ashland, OR); 2-D contour plots were exported as scalable vector graphic (svg) files to CorelDraw2017. The median level of expression of each marker was used to assign a phenotypic identity to each cluster. Heat maps were generated using Morpheus software (www.broadinstitute.org) and exported to CorelDraw2017 as svg files. Principal component analysis (PCA) was performed using PAST software (https://palaeo-electronica.org/2001_1/past/issue1_01.htm).

## Results

**Figure 1:**
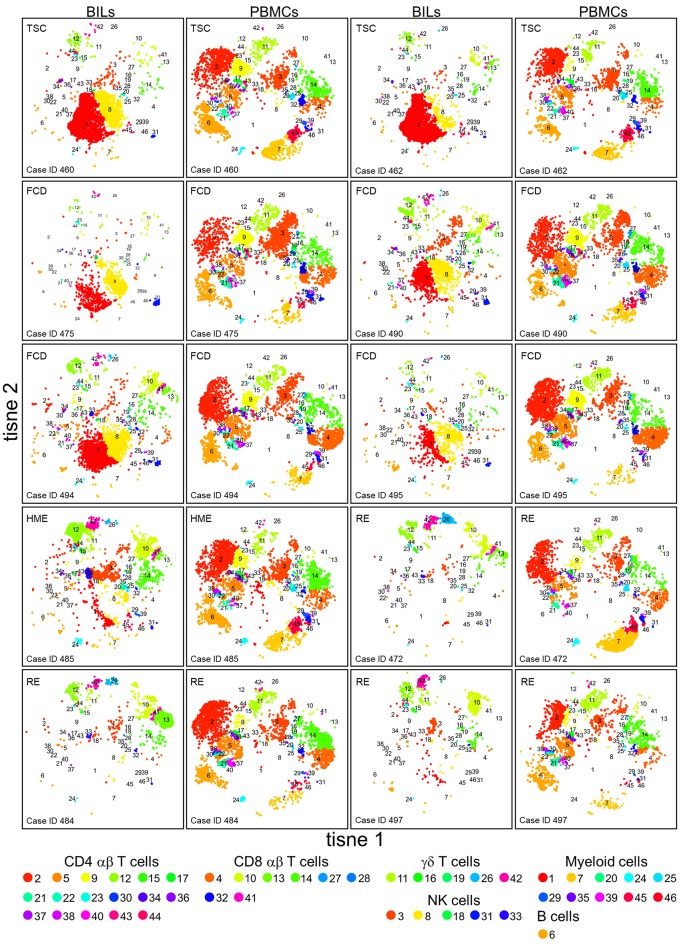
tSNE plots showing the relative number of different immune cells in BILs and PBMCs from the pediatric epilepsy surgeries. The expression of 20 immune cell markers was analyzed by CyTOF. To define subsets of CD45^+^ cells in each BIL and PBMC population, the entire high dimensional dataset (comprising 20 FCS files) was converted into a matrix of pair-wise similarities by implementing the t-based stochastic neighbor embedding (t-SNE) algorithm, followed by a density-based clustering method (ClusterX). The clusters were assigned as either T cells, NK cells, myeloid cells, or B cells based on the median expression values of specific immune cell markers (CD3, CD4, CD8, TCR γδ, CD11b, CD56, and CD19).

**Figure 2:**
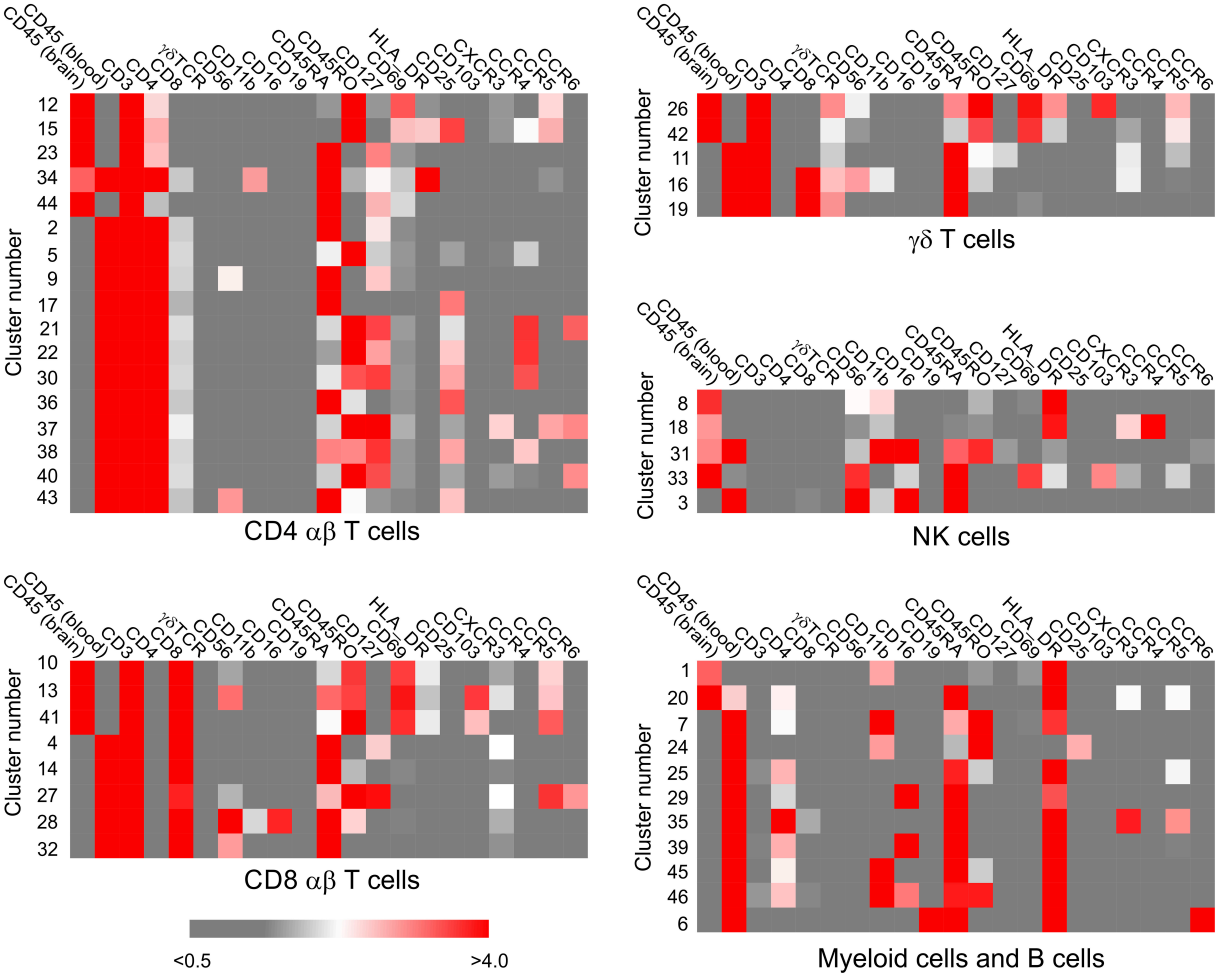
Assignment of immune cell phenotypes. The median expression values of 19 immune cell markers, calculated by the Cytofit software, were used to assign a phenotype to each cluster of CD45^+^ cells (Table S2). The data were first separated into CD3^+^ and CD3^−^ clusters, and the CD3^+^ populations were further subdivided into CD4^+^, CD8^+^, γδ subsets. The CD3^−^ populations were categorized as myeloid, natural killer cell, or B cell based on the expression of CD56 and CD19. Heat maps generated from the median expression values included all the markers that were expressed on cells in the CD3^+^ CD4^+^, CD3^+^ CD8^+^, CD3^+^ γδ^+^, CD3^−^ CD56^+^, CD3^−^ CD19^+/−^ clusters, respectively. The median expression value of the two different CD45 antibody metal conjugates used to stain the PBMC and BIL fractions reflects the relative number of PBMCs and BILs in each cluster.

PBMCs and BILs isolated from 10 pediatric epilepsy cases were analyzed by CyTOF using a panel of antibodies designed to identify populations of adaptive lymphoid cells, innate lymphoid cells, and myeloid cells. Implementing the mass cytometry data analysis pipeline developed by Chen et al. (21) generated 46 clusters, corresponding to putatively distinct populations of CD45^+^ cells (Figure 1). Clusters were classified as T cells, NK cells, and myeloid cells based on the relative expression of 19 immune cell markers. Immune cell clusters were divided into a CD3^+^ (*n* = 30, median CD3 expression values of 4.648–6.283) and a CD3^−^ group (*n* = 16, median expression values of 0.001–0.81). The CD3^+^ group was subdivided into subsets of CD4, CD8, and γδ T cells based on the level of expression of these three phenotypic markers (Figure 2). The CD3^−^ group was further divided into five NK cell subsets, ten myeloid and one B cell population based on the expression of CD56 and CD19 (Figure 2; Table S2).

**Figure 3:**
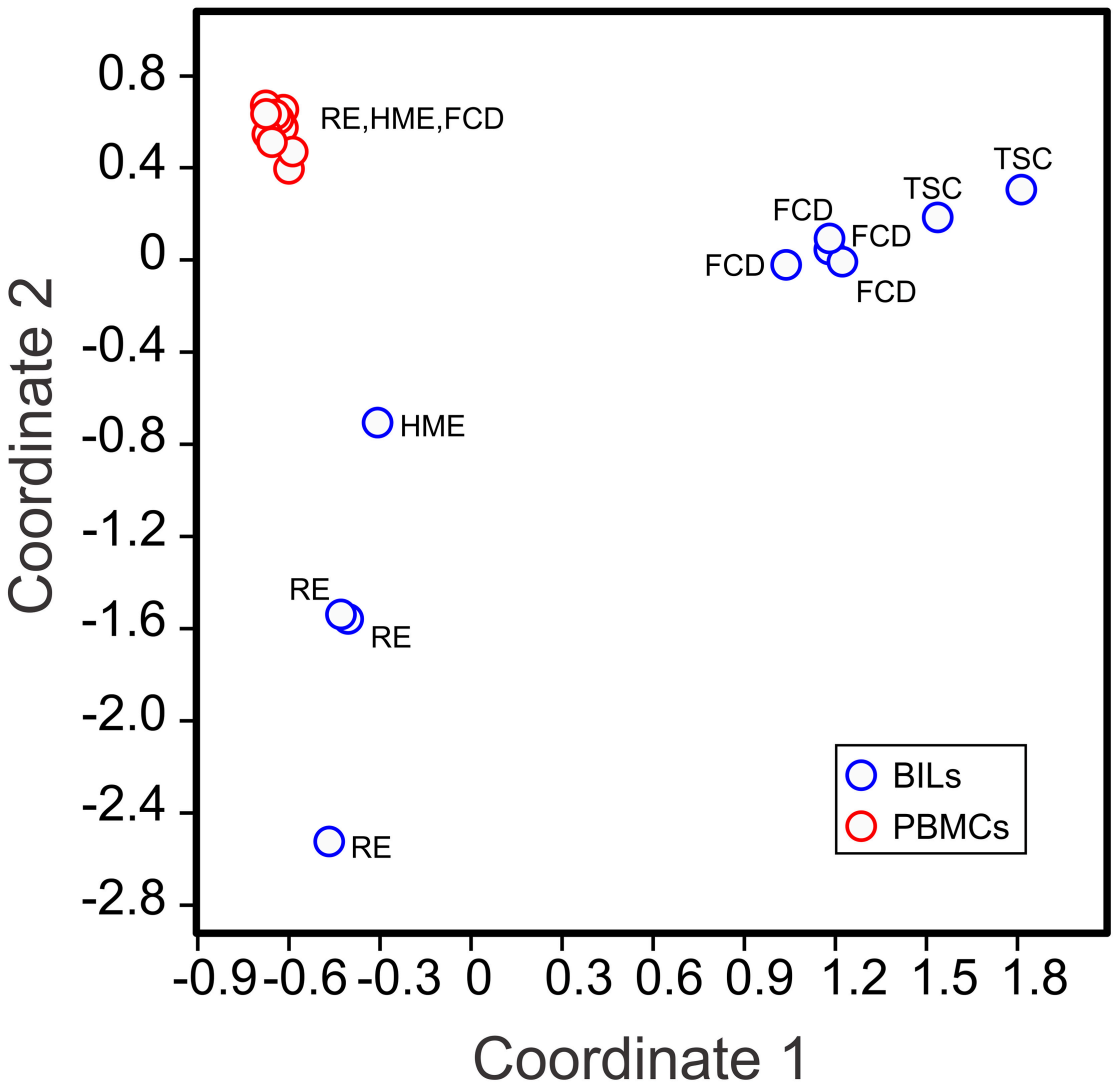
Clustering of BILs and PBMCs from pediatric epilepsy surgeries. The relative number of cells in each cluster in each sample (as a percentage of the total number of CD45^+^ cells analyzed in each sample) was used to implement a principal component analysis (PCA). The first two components could account for 65.72% of the total variance in the data set. The 2D PCA plot shows that the BIL fractions are clearly different from the corresponding PBMCs, and that BILs from the RE and HME cases are distinct from those from the FCD and TSC cases.

**Figure 4:**
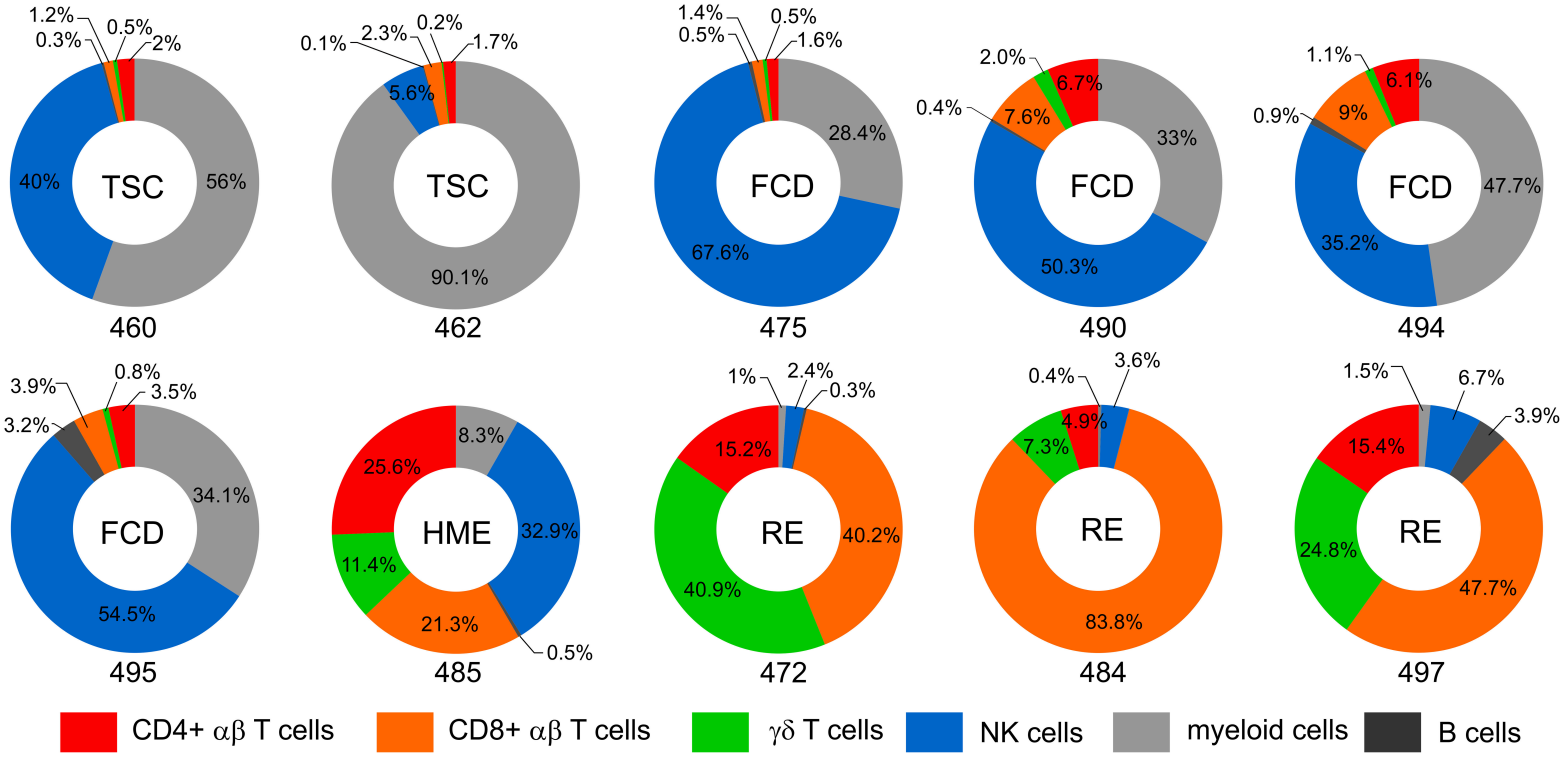
Immune cell profiles of BILs. Donut plots show the relative number of lymphoid cells, myeloid cells and B cells in the BIL fractions isolated from resected brain tissue. Lymphoid cells are divided into CD4, CD8, and γδ T cells and NK cells. Each chart corresponds to one of the surgery cases, and are arranged according to the clinical diagnosis (TSC: 460, 462; FCD: 475, 490, 494, 495; HME: 485; RE: 472, 484, 497). The data are presented as percentages of the number of CD45^+^ cells in each BIL fraction.

Visual inspection of the t-SNE plots (Figure 1) showed that there were clear differences between the BILs from each surgical case compared with the corresponding PBMCs. On the other hand, the profiles of BILs from the two TSC (Case IDs 460 and 462) and the four FCD cases (Case IDs 475, 490, 494, and 495) appeared to be very similar and distinct from the three RE cases (Case IDs 472, 484, and 497), and dissimilar from the HME (Case ID 485), which appeared more similar to the RE cases. Principal components analysis based on the relative abundance of all of the clusters in each sample (percentages of CD45^+^ cells) confirmed this observation, and also showed that the immune cell profiles of PBMCs from all of the cases were very similar (Figure 3). From the magnitude of the PCA loading values (Table S3), Clusters 1 and 8 accounted for the largest amount of variance in the first component, thus, the relative numbers of NK and myeloid cells in the BIL fractions appears to explain in large measure the observed difference between the TSC and FCD BILS compared with the RE and HME BILs. As summarized in Figure 4, CD45^+^ cells from the FCD and TSC brain tissue specimens comprised far more NK cells and myeloid cells than CD45^+^ RE BILs, which in agreement with previous studies (5, 6, 20, 23), were predominantly CD8^+^ αβ T and γδ cells, The HME BILs comprised approximately equal numbers of T cells, NK cells and myeloid cells.

**Figure 5:**
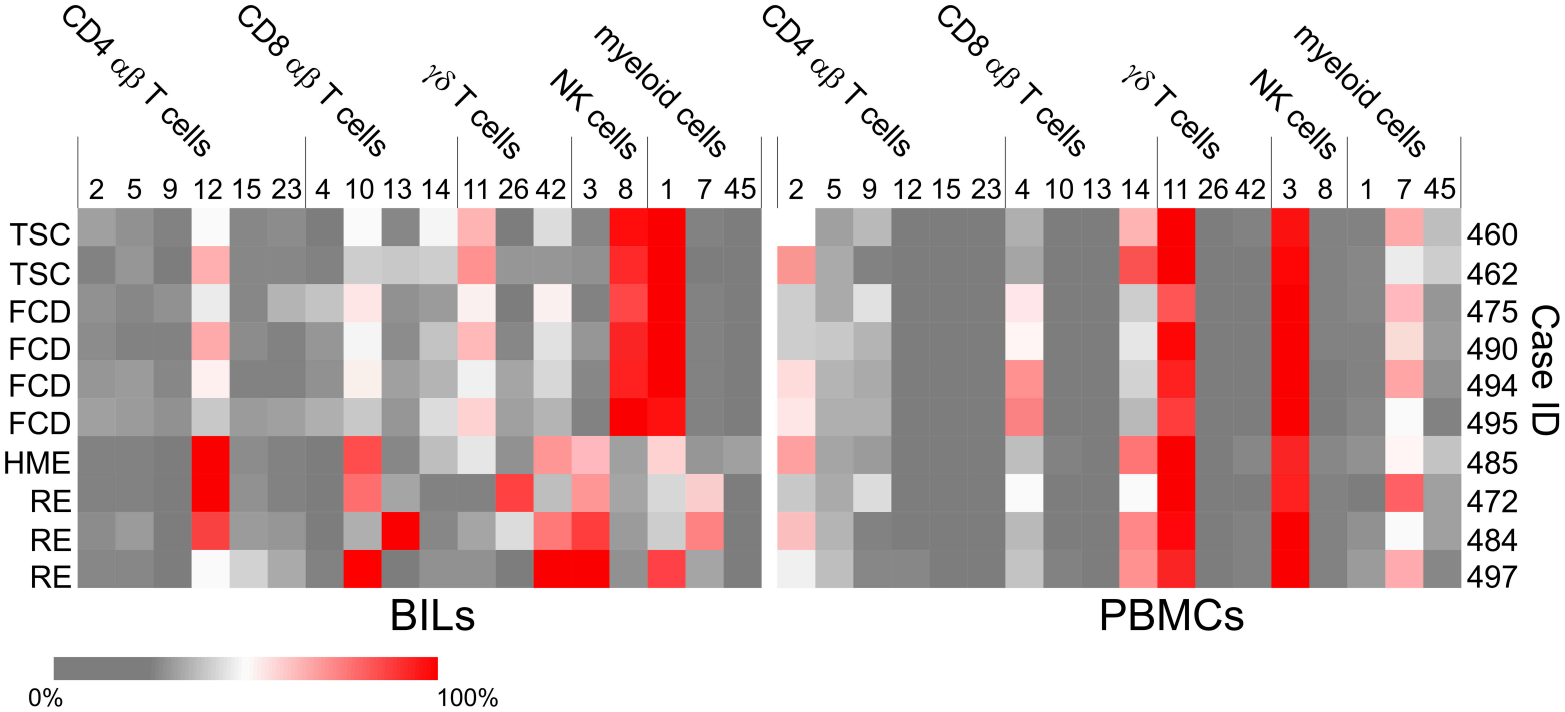
Heat maps showing the subsets of T cells, NK and myeloid cells that account for the majority of lymphoid and myeloid cells in each BILs and PBMC fraction. The heat maps show the relative abundance of each cluster as a percentage of the total number of CD4 αβ T cells, CD8 αβ T cells, γδ T cells, NK cells or myeloid cells, respectively in each BIL and PBMC fraction (Table S4).

Three populations of myeloid cells (Clusters 1, 7, and 45) (Figure 2) made up >50 percent of all of the myeloid cells in all of the samples (Figure 5; Table S4). The Cluster 1 myeloid population comprised the vast majority of CD11b^+^ cells in the BIL fractions from the FCD and TSC cases (Figure 5), whereas Cluster 7 CD11b^+^ myeloid cells were more abundant in two of the three RE BIL fractions (Case IDs 472 and 484), and in all of the PBMCs (Figure 5). The only other marker that defined the Cluster 1 population was HLA-DR, thus it was not possible to assign a definitive phenotype to cells in this population, but they are likely to be macrophages. Likewise, Cluster 7 cells, which are CD3^−^ CD4^lo^ could constitute a dendritic cell or monocyte population, but additional markers are also required to adequately define this population.

Cluster 3 NK cells are CD56^+^ CD45RA^+^ HLA-DR^−^ CD11b^+^ CD16^+^ (Figure 2), and constituted the major population of NK cells in the blood from all of the cases (Figure 5). This subset also comprised the majority of NK cells that were found in BILs from the resected RE and HME brain tissue (Figure 5). By contrast, Cluster 8 NK cells, defined as CD56^+^ CD45RA^−^ HLA-DR^+^ CD11b^−^ CD16^−^ (Figure 2) comprised 86–96 percent of the NK cells isolated from the FCD and TSC brain tissue (Figure 5; Table S4). We confirmed the existence of Cluster 3 and 8 NK cell populations by manually curating the original FCS files from FCD case 490 using the FlowJO software package (Figure S1A).

Six subpopulations of CD4^+^ αβ T cells (Clusters 2, 5, 9, 12, 15, and 23), four of CD8^+^ αβ T cells (Clusters 4, 10, 13, and 14), and three subsets of γδ T cells (Clusters 11, 26, and 42) accounted for over 80% of the CD3^+^ T cells in all of the BIL and PBMC samples, although there were marked differences in the proportion of the different T cell subsets between the BIL and PBMC samples (Figure 5; Table S4). Clusters 2, 9 and 23 CD4^+^ T cells constitute naïve T cell populations (CD45RA^+^ CD45RO^−^ CD127^int^) (Figure 2); different median levels of expression of CD4 and CD127 appear to account for the generation of two clusters of naïve conventional T cells (Clusters 2 and 23), whereas expression of CD56 by Cluster 9 CD45RA^+^CD45RO^−^CD4^+^ T cells indicates that they may be unconventional NKT cells.

Clusters 5 and 15 define two different subpopulations of antigen-experienced CCR4^+^ CD4^+^ T cells (Figure 2). Based on the expression of CD25, the interleukin-2 receptor α chain (IL-2R) and lack of CD127, the interleukin-7 receptor α chain (IL-7R), Cluster 15 cells appear to be conventional regulatory T cells (Tregs) (24), and were found almost exclusively in the BIL fractions (Figure 5). Additional minor populations of CD45RO^+^ CD25^+^ CCR4^+^ CD4 T cells (Clusters 21, 22, 30, and 38) found predominantly in the blood may also be Tregs (Figure 2). Cluster 5 CD4 cells were found in both the brain and blood, and may be both IL-7 and IL-2 dependent (Figures 2, 5). Cluster 12 defined an activated effector memory population of CD4 T cells (CD45RO^+^ HLA-DR^+^ CD69^+^) (Figure 2) that was found in all of the BIL fractions (Figure 5). These cells also expressed the chemokine receptors CXCR3 and CCR5 thus may have trafficked to the epileptogenic brain area that was resected, possibly due to local inflammation (25).

Cluster 4 CD8 T cells were primarily found in the PBMC fractions and appear to correspond to an activated unprimed subtype (CD45RA^+^ CD127^+^) since they also expressed the chemokine receptor CXCR3 (26). A CD45RA^+^ CD45RO^lo^ subpopulation (Cluster 14) made up most of the remaining CD8 T cells in the blood (Figures 2, 5; Table S4). Cells in Clusters 10 and 13 appear to be activated (CD69^+^ HLA-DR^+^) effector memory subtypes, and were found almost exclusively in the brain (Figures 2, 5). Based on the marker panel used in this study, the only difference between these two clusters was the expression of the resident memory marker CD103. Fewer Cluster 13 CD8^+^ resident memory T cells (T_RM_) were found in the brain compared with Cluster 10 T cells with one exception, Case ID 484, a RE patient. In agreement with previous work (27), the majority of the CD8^+^ T cells isolated from resected brain tissue from this patient were T_RM_ cells. Likewise, the majority of γδ T cells isolated from a second RE case (Case ID 472) were T_RM_ cells (Cluster 26, Figure 5) (27). Cluster 26 and Cluster 42 γδ T cells expressed the activation markers CD69 and HLA-DR and were almost exclusively found the brain (Figures 2, 5). These two activated subsets comprised 88–98 percent of the γδ T cells in the RE BIL fractions, whereas a third subset, Cluster 11, a CCR5^+^ CXCR3^+^ effector memory population (CD45RA^+^ CD45RO^int^ CD127^+^) contributed up 70 percent of the γδ T cells in the other cases (Table S4). The Cluster 11 subset also made up the vast majority of γδ T cells in the blood from all of the cases (Figure 5). We verified the existence of all of the major T cell populations defined by the ClusterX clustering algorithm by manually curating several of the original FCS files (Figures S1B–D).

**Figure 6:**
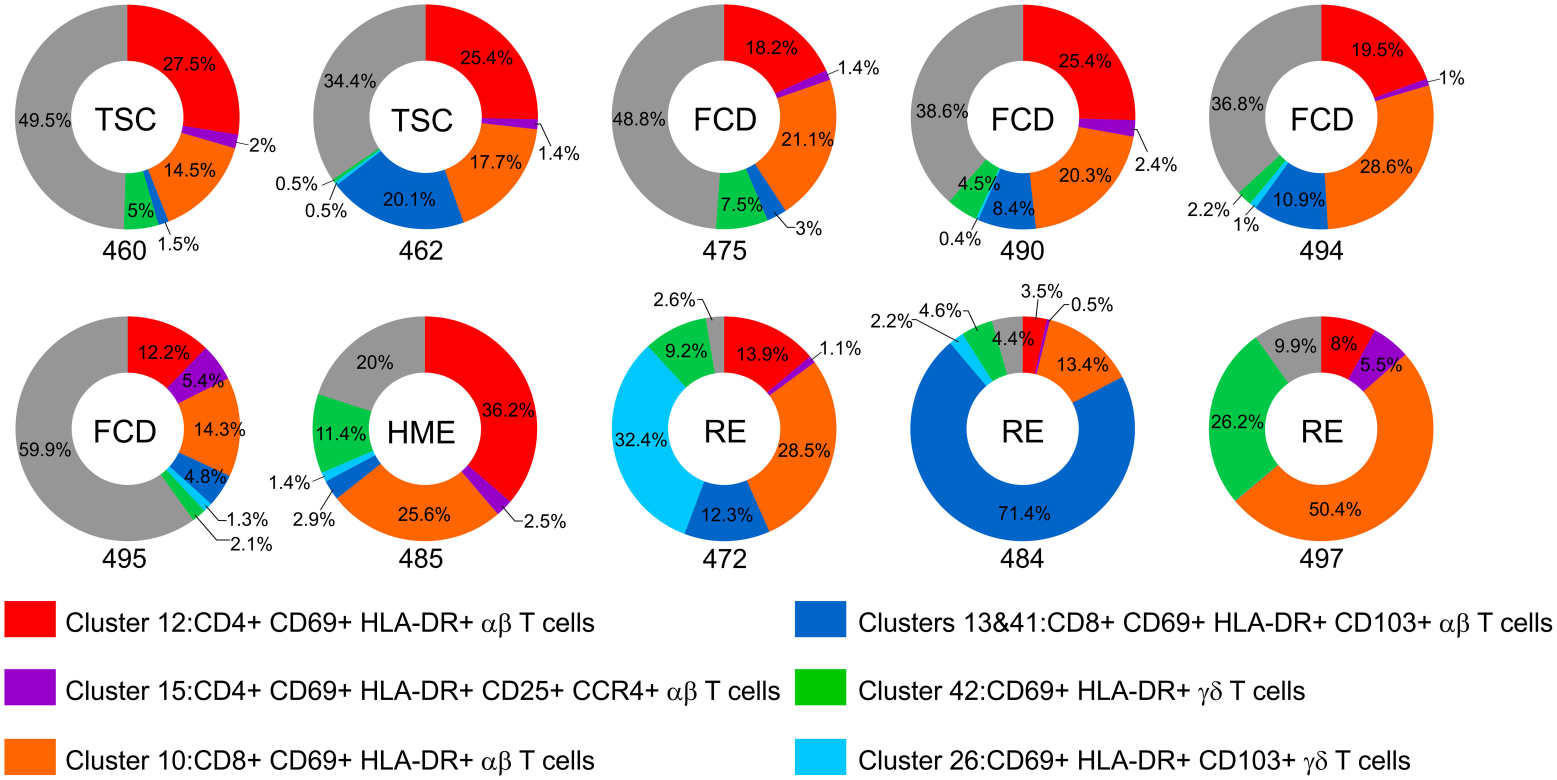
The proportion of activated T cells in the BIL fractions. Donut plots show the relative number of CD69^+^ HLA-DR^+^ CD4, CD8, and γδ T cells as percentage of the CD3^+^ cells in each BIL fraction. Each chart corresponds to one of the surgery cases, and are arranged according to the clinical diagnosis (TSC: 460, 462; FCD: 475, 490, 494, 495; HME: 485; RE: 472, 484, 497). The different activated subsets are color coded; the gray area denotes the percentage of T cells that did not express activation markers.

The donut plots in Figure 6 summarize the proportion of CD69^+^ HLA-DR^+^ T cells found in each of the BIL fractions from the 10 surgery cases. At least 90 percent of the T cells isolated from the RE cases and 80 percent of the T cells from the HME case were activated. Even though T cells made up only a small fraction of the CD45^+^ cells isolated from the TSC and FCD cases, 40–66 percent of the T cells were also activated at the time of surgery.

**Figure 7:**
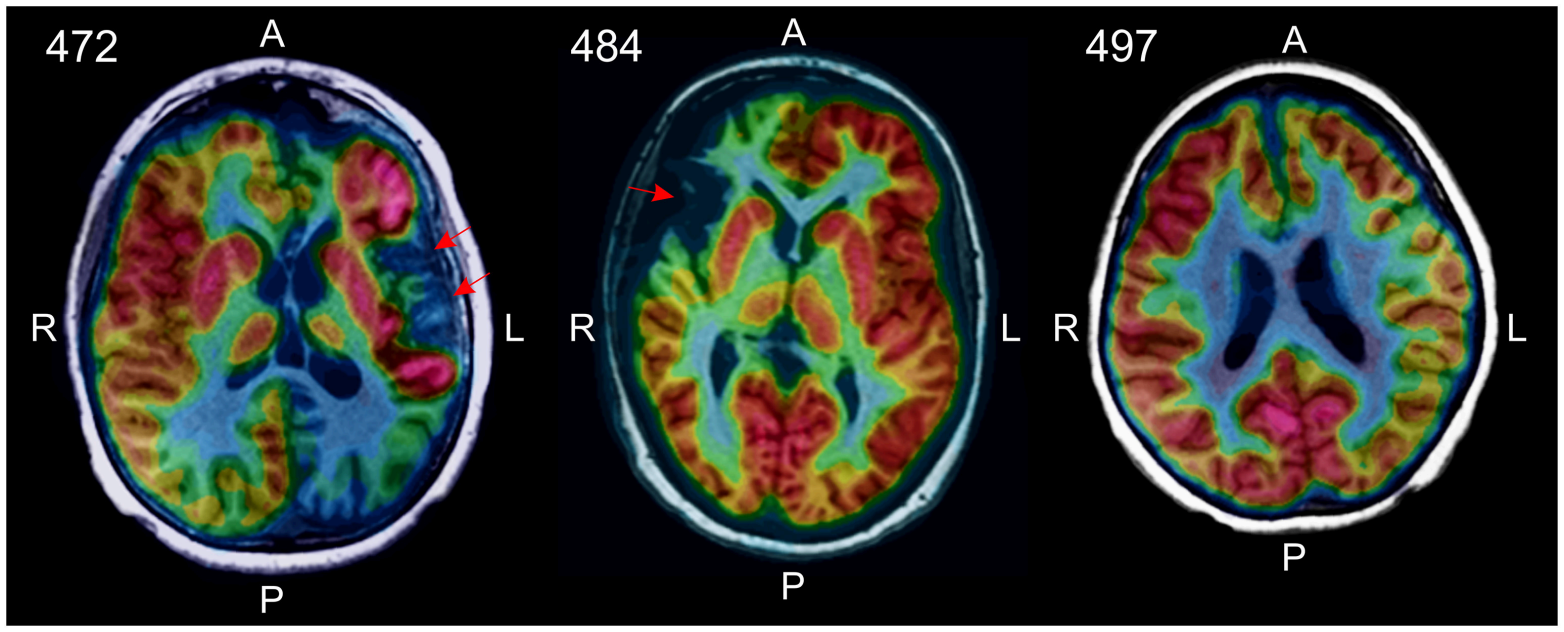
[^18^F]-deoxyglucose positron emission tomography (FDG-PET) identified areas of atrophy in RE cases 472 and 484 (red arrows). In RE case 497 there were no obvious signs of atrophy in the affected hemisphere, rather decreased glucose metabolism over the entire hemisphere was apparent.

The absence of CD8^+^ T_RM_ cells in the BIL fraction from RE case ID 497 suggested that the disease may not have progressed as far in this patient compared with the other two RE cases in the study cohort (Cases IDs 472 and 484). A [^18^F]-deoxyglucose positron emission tomography (FDG-PET) brain scan of the patient made before the surgery showed hypometabolism extending over the entire affected hemisphere, with no definitive areas of atrophy. In the other two RE cases, areas of atrophy were clearly visible (Figure 7). Case ID 497 was the youngest of the three RE patients at the time of seizure onset and underwent surgery after the shortest time following onset of seizures (Table S1).

## Discussion

We have used CyTOF to gain a better understanding of the involvement of peripheral immune cells in intractable epilepsy in children. We implemented an unsupervised clustering method to resolve the resulting high dimensional data into the main subtypes of adaptive and innate lymphoid cells present in resected epileptogenic brain tissue. Our small cohort study, and other recent work (28) implicate cellular immunity not only in RE, but in intractable epilepsy in children where an infection or immune disorder is not suspected. With the caveat that we only analyzed a limited number of surgery cases, we found a clear difference in the relative number of innate vs. adaptive peripheral immune cells in fractions of CD45^+^ cells isolated from resected TSC and FCD brain tissue compared with involved RE brain tissue. The FCD and TSC BIL fractions were dominated by a single subset of NK cells (Cluster 8) and a population of myeloid cells (Cluster 1) that are likely to be macrophages, possibly classically activated pro-inflammatory M1 macrophages. Adding CD38 and CD163 antibodies to the staining panel will allow us to distinguish between M1 and M2 macrophages, respectively (29, 30). In an animal seizure model, infiltrating monocytes were shown to exacerbate neuronal damage (31), indicating that the abundant myeloid population that we found in the FCD and TSC BIL fractions may be pathologically relevant.

Most NK cells in the blood express a low level of CD56 (CD56^dim^) and are CD16^+^, thus can engage in antibody-dependent cell-mediated cytotoxicity by binding antibody-coated target cells via the low affinity Fcγ receptor III (CD16) (32). Fewer circulating NK cells express a high level of CD56 (CD56^bright^); these cells generally lack CD16, but can produce pro-or anti-inflammatory cytokines (32). CD56^bright^ NK cells are considered to be more immature than CD56^dim^ NK cells in that they mostly do not express killer cell immunoglobulin-like receptors (KIRs) (33). Unlike CD56^dim^ NK cells they express CD62L (L-selectin) and CD197 (CCR7), cell surface molecules that facilitate homing to secondary lymph nodes (32). CD56^dim^ NK cells express KIRs and CD57 and are considered to be intrinsically more cytotoxic (32, 33). The most abundant NK cells that we found in the blood of all of the patients in our study cohort presumably correspond to CD56^dim^CD16^+^ NK cells (Cluster 3). This subset also comprised the majority of NK cells present in the BIL fractions from the RE and HME patients (Figure 5). Adding CD62L, CD197, and CD57 to the marker panel will clarify the phenotype of Cluster 3 NK cells.

By comparison to the NK cells in the PBMC fractions, the majority of NK cells found in the FCD and TSC BIL fractions expressed a lower level of CD56 suggesting that they correspond to a CD56^dim^ subset (Cluster 8) (Figure 2, Figure S1A). Unlike canonical CD56^dim^ NK cells, Cluster 8 cells did not express CD16, however populations of CD56^dim^ NK cells expressing little or no CD16 have been previously described (34). Lack of CD16 may be due to the fact that the Cluster 8 NK cells were highly activated; it has been reported that the Fcγ receptor III is proteolytically cleaved in degranulating CD56^dim^ NK cells (35). In contrast to Cluster 3 NK cells, Cluster 8 NK cells also expressed HLA-DR (Figure 2, Figure S1A), which suggests that they may be adaptive NK cells (32, 33). A third population of NK cells (Cluster 33), only found in appreciable numbers in BILs from the HME case (Case ID 485) expressed CD69 along with CCR5, CXCR3, and CD103 suggesting that this cluster may correspond to an activated resident subset (32).

BIL fractions from the RE cases were predominantly CD8 αβ and γδ effector memory and resident memory T cells with fewer effector memory CD4 T cells, confirming previous work (20, 27). We and others have shown that T cells found in RE brain are clonally focused (7–10), which strongly implicates an antigen-driven adaptive immune response in the etiology of RE, and is consonant with the idea that RE is an autoimmune disease (36). Finding T_RM_ CD8 αβ and γδ T cells in affected RE brain tissue indicates that an earlier immune response had occurred that left a resident memory population in place. Compared with RE cases 472 and 484 however, no T_RM_ cells were present in the BILs isolated from RE case 497, suggesting that this case may represent an early stage of the disease. In support of this idea, brain atrophy, a feature of late phases of the disease (4), was not evident in an FDG-PET brain scan of RE case 497, but was in RE cases 472 and 484 (Figure 7).

In pathological specimens from FCD and TSC surgeries scattered T cells have been previously observed (16, 17), and in our study we found activated CD4, CD8, and γδ T cells in the BIL fractions from the FCD and TSC cases (Figure 6). Determining the clonal diversity of these T cells by sequencing Vβ chain third complementarity determining regions may indicate whether they are unspecific bystanders or presage an adaptive response against self-antigens. Binding of self-peptides would however depend on the MHC alleles carried by the individual (37–39). The proportion of activated T cells in the single HME case (Case ID 485), which exceeded the number of NK cells (Figure 4), may indicate that an autoimmune response had already occurred in this very young patient who suffered from severe seizures. Future recall assays in which T cells isolated from resected brain tissue are cocultured with autologous neurons and glia differentiated from patient-derived induced pluripotent stems cells will directly answer whether the T cells are autoreactive.

The immune cell profiles of peripheral blood from all of the cases comprised both naïve and antigen experienced T cells. γδ T cells that were CD127^+^, CD45RO^+^, CXCR3^+^, and CCR5^+^ comprised the majority of γδ T cells in all of the PBMC samples, as well as in the FCD and TSC BILs (Cluster 11; Figures 2, 5, Figure S1D). They likely express Vδ2/Vγ9 TCR receptors (40) since Vδ2/Vγ9 γδ T cells make up a large fraction of circulating γδ T cells (41). Expression of CXCR3 and CCR5 would allow these γδ T cells to access sites of inflammation (26). CXCR3 expression by naïve CD45RA^+^ CD8 T cells (Cluster 4; Figure 2, Figure S1C) indicates that they are activated (42), and that development into effector T cells may be enhanced (43, 44). Whether the presence of these T cell subsets in the blood is directly related to inflammation in the brain remains to be determined. In future work we plan to compare the immune cell profiles of blood collected at the time of surgery and after recovery from surgery. Removal of epileptogenic brain tissue, the potential source of inflammation may change the relative frequency of specific circulating T cell subsets. Such subsets could potentially be used as biomarkers to assess the extent brain inflammation in children presenting with intractable epilepsy.

The distribution of the two most abundant CCR4^+^ CD4 T cell subsets (Clusters 5 and 15) differed between the blood and brain. Cluster 15 cells were almost exclusively found in BIL fractions, and appear to be activated effector memory-like Tregs (CD25^+^ CD45RO^+^CD69^+^HLA-DR^+^) (Figures 2, 5, Figure S1B). Tregs are also defined by expression of the transcription factor FOXP3 (24); adding this intracellular marker to the CyTOF panel will confirm the identity of these CD4 T cells. In a cohort of pediatric epilepsy surgery patients, a negative correlation between seizure frequency and the relative number of Tregs in resected epileptogenic brain was recently reported (28). In our study cohort, the relative number of Cluster 15 Tregs cells ranged from 2.61 to 31.53 percent of CD4 T cells in the BIL fractions (Table S4). However, the limited number of self-reports of seizure activity available do not allow us to draw any conclusions about the number of seizures and the percent Tregs in our patient cohort. Cluster 5 CD45RO^+^CCR4^+^ CD4 T cells (Figure 2, Figure S1B), which express both IL-2Rs and IL-7Rs, were found in both BIL and PBMC fractions, and may correspond to a subset of CD4 T cells that express CD25 and CD127 and low levels of FOXP3, and are not suppressive *in vitro* (24). Adding the FOXP3 Ab to the marker panel may help clarify the identity of this subset of CD4 T cells.

Extensive animal studies have shown that inflammation in the brain is both a cause and a consequence of seizure activity (45). Attention has focused on the innate response of brain resident microglia and astrocytes to seizures and on the pro-inflammatory molecules produced by these cells as drivers of epileptogenesis (46–48). In seizure models, chemokines and cytokines produced by activated microglia and astrocytes can cause blood brain barrier leakage, and can attract peripheral lymphoid and myeloid cells to the brain (31, 49–51). Our data suggest that this could be happening in children with chronic pharmacoresistant seizures. Intractable epilepsy might be viewed as a “sterile” infection in which T cells and other immune cells traffic to epileptogenic areas of the cerebral cortex in response to local seizure-driven inflammation. Activated T cells and NK cells for example could permanently alter brain circuitry by directly or indirectly damaging neurons (52–55). Adjunctive treatments that block the recruitment of pro-inflammatory peripheral lymphoid and myeloid cells to the brain (56–61) may therefore be therapeutically beneficial.

## Author Contributions

GO designed the study, analyzed the data, and wrote the paper. AG assisted with study design and performed the CyTOF. AM assisted with analysis of the data. JC and SR coordinated collection of surgical specimens and prepared BILs and PBMCs. RP assisted with study design and helped draft the paper. NS provided FDG-PET results. GM supported the project. AF provided support for the work, and provided surgical specimens and patient information.

### Conflict of Interest Statement

The authors declare that the research was conducted in the absence of any commercial or financial relationships that could be construed as a potential conflict of interest.

## References

[1] Guerrini R. Epixlepsy in children. Lancet (2006) 367:499–524. 10.1016/S0140-6736(06)68182-8 16473127

[2] Berg AT Kelly MM. Defining intractability: comparisons among published definitions. Epilepsia (2006) 47:431–6. 10.1111/j.1528-1167.2006.00440.x 16499772

[3] Varadkar S Bien CG Kruse CA Jensen FE Bauer J Pardo CA Rasmussen's encephalitis: clinical features, pathobiology, and treatment advances. Lancet Neurol. (2014) 13:195–205. 10.1016/S1474-4422(13)70260-6 24457189 PMC4005780

[4] Bien CG Urbach H Deckert M Schramm J Wiestler OD Lassmann H. Diagnosis and staging of Rasmussen's encephalitis by serial MRI and histopathology. Neurology (2002) 58:250–7. 10.1212/WNL.58.2.250 11805253

[5] Prayson RA Frater JL. Rasmussen encephalitis: a clinicopathologic and immunohistochemical study of seven patients. Am J Clin Pathol. (2002) 117:776–82. 10.1309/AD8R-560C-4V11-C5E2 12090428

[6] Pardo CA Vining EP Guo L Skolasky RL Carson BS Freeman JM. The pathology of Rasmussen syndrome: stages of cortical involvement and neuropathological studies in 45 hemispherectomies. Epilepsia (2004) 45:516–26. 10.1111/j.0013-9580.2004.33103.x 15101833

[7] Schwab N Bien CG Waschbisch A Becker A Vince GH Dornmair K. CD8+ T-cell clones dominate brain infiltrates in Rasmussen encephalitis and persist in the periphery. Brain (2009) 132:1236–46. 10.1093/brain/awp003 19179379

[8] Schneider-Hohendorf T Mohan H Bien CG Breuer J Becker A Gorlich D. CD8(+) T-cell pathogenicity in Rasmussen encephalitis elucidated by large-scale T-cell receptor sequencing. Nat Commun. (2016) 7:11153. 10.1038/ncomms11153 27040081 PMC4822013

[9] Dandekar S Wijesuriya H Geiger T Hamm D Mathern GW Owens GC. Shared HLA CLASS I and II alleles and clonally restricted public and private brain-infiltrating alphabeta T cells in a cohort of rasmussen encephalitis surgery patients. Front Immunol. (2016) 7:608. 10.3389/fimmu.2016.00608 28066418 PMC5165278

[10] Al Nimer F Jelcic I Kempf C Pieper T Budka H Sospedra M. Phenotypic and functional complexity of brain-infiltrating T cells in Rasmussen encephalitis. Neurol Neuroimmunol Neuroinflamm. (2018) 5:e419. 10.1212/NXI.0000000000000419 29259996 PMC5733246

[11] Martin KR Zhou W Bowman MJ Shih J Au KS Dittenhafer-Reed KE. The genomic landscape of tuberous sclerosis complex. Nat Commun. (2017) 8:15816. 10.1038/ncomms15816 28643795 PMC5481739

[12] Polchi A Magini A Meo DD Tancini B Emiliani C. mTOR signaling and neural stem cells: the tuberous sclerosis complex model. Int J Mol Sci. (2017) 19:E1474. 10.3390/ijms19051474 29772672 PMC5983755

[13] Mirzaa GM Campbell CD Solovieff N Goold C Jansen LA Menon S. Association of MTOR mutations with developmental brain disorders, including megalencephaly, focal cortical dysplasia, and pigmentary mosaicism. JAMA Neurol. (2016) 73:836–45. 10.1001/jamaneurol.2016.0363 27159400 PMC4979321

[14] D'Gama AM Woodworth MB Hossain AA Bizzotto S Hatem NE LaCoursiere CM. Somatic mutations activating the mTOR pathway in dorsal telencephalic progenitors cause a continuum of cortical dysplasias. Cell Rep. (2017) 21:3754–766. 10.1016/j.celrep.2017.11.106 29281825 PMC5752134

[15] Alcantara D Timms AE Gripp K Baker L Park K Collins S. Mutations of AKT3 are associated with a wide spectrum of developmental disorders including extreme megalencephaly. Brain (2017) 140:2610–22. 10.1093/brain/awx203 28969385 PMC6080423

[16] Boer K Jansen F Nellist M Redeker S van den Ouweland AM Spliet WG. Inflammatory processes in cortical tubers and subependymal giant cell tumors of tuberous sclerosis complex. Epilepsy Res. (2008) 78:7–21. 10.1016/j.eplepsyres.2007.10.002 18023148

[17] Iyer A Zurolo E Spliet WG van Rijen PC Baayen JC Gorter JA. Evaluation of the innate and adaptive immunity in type I and type II focal cortical dysplasias. Epilepsia (2010) 51:1763–73. 10.1111/j.1528-1167.2010.02547.x 20345941

[18] Boer K Crino PB Gorter JA Nellist M Jansen FE Spliet WG. Gene expression analysis of tuberous sclerosis complex cortical tubers reveals increased expression of adhesion and inflammatory factors. Brain Pathol. (2010) 20:704–19. 10.1111/j.1750-3639.2009.00341.x 19912235 PMC2888867

[19] Prabowo AS Anink JJ Lammens M Nellist M van den Ouweland AM Adle-Biassette H. Fetal brain lesions in tuberous sclerosis complex: TORC1 activation and inflammation. Brain Pathol. (2013) 23:45–59. 10.1111/j.1750-3639.2012.00616.x 22805177 PMC3518755

[20] Owens GC Erickson KL Malone CC Pan C Huynh MN Chang JW. Evidence for the involvement of gamma delta T cells in the immune response in Rasmussen encephalitis. J Neuroinflammation (2015) 12:134. 10.1186/s12974-015-0352-2 26186920 PMC4506578

[21] Chen TJ Kotecha N. Cytobank: providing an analytics platform for community cytometry data analysis and collaboration. Curr Top Microbiol Immunol. (2014) 377:127–57. 10.1007/82_2014_364 24590675

[22] Chen H Lau MC Wong MT Newell EW Poidinger M Chen J. Cytofkit: a bioconductor package for an integrated mass cytometry data analysis pipeline. PLoS Comput Biol. (2016) 12:e1005112. 10.1371/journal.pcbi.1005112 27662185 PMC5035035

[23] Bien CG Bauer J Deckwerth TL Wiendl H Deckert M Wiestler OD. Destruction of neurons by cytotoxic T cells: a new pathogenic mechanism in Rasmussen's encephalitis. Ann Neurol. (2002) 51:311–8. 10.1002/ana.10100 11891826

[24] Sakaguchi S Miyara M Costantino CM Hafler DA. FOXP3+ regulatory T cells in the human immune system. Nat Rev Immunol. (2010) 10:490–500. 10.1038/nri2785 20559327

[25] Trebst C Staugaitis SM Tucky B Wei T Suzuki K Aldape KD. Chemokine receptors on infiltrating leucocytes in inflammatory pathologies of the central nervous system (CNS). Neuropathol Appl Neurobiol. (2003) 29:584–95. 10.1046/j.0305-1846.2003.00507.x 14636165

[26] Groom JR Luster AD. CXCR3 in T cell function. Exp Cell Res. (2011) 317:620–31. 10.1016/j.yexcr.2010.12.017 21376175 PMC3065205

[27] Owens GC Chang JW Huynh MN Chirwa T Vinters HV Mathern GW. Evidence for resident memory T cells in rasmussen encephalitis. Front Immunol. (2016) 7:64. 10.3389/fimmu.2016.00064 26941743 PMC4763066

[28] Xu D Robinson AP Ishii T Duncan DS Alden TD Goings GE. Peripherally derived T regulatory and gammadelta T cells have opposing roles in the pathogenesis of intractable pediatric epilepsy. J Exp Med. (2018) 215:1169–86. 10.1084/jem.20171285 29487082 PMC5881465

[29] Zwadlo G Voegeli R Schulze Osthoff K Sorg C A monoclonal antibody to a novel differentiation antigen on human macrophages associated with the down-regulatory phase of the inflammatory process. Exp Cell Biol. (1987) 55:295–304. 3450546 10.1159/000163432

[30] Amici SA Young NA Narvaez-Miranda J Jablonski KA Arcos J Rosas L. CD38 is robustly induced in human macrophages and monocytes in inflammatory conditions. Front Immunol. (2018) 9:1593. 10.3389/fimmu.2018.01593 30042766 PMC6048227

[31] Varvel NH Neher JJ Bosch A Wang W Ransohoff RM Miller RJ. Infiltrating monocytes promote brain inflammation and exacerbate neuronal damage after status epilepticus. Proc Natl Acad Sci USA. (2016) 113:E5665–74. 10.1073/pnas.1604263113 27601660 PMC5035862

[32] Cichicki F Schlums H Theorell J Tesi B Miller JS Ljunggren HG. Diversification and functional specialization of human NK cell subsets. Curr Top Microbiol Immunol. (2016) 395:63–94. 10.1007/82_2015_487 26472216

[33] Freud AG Mundy-Bosse BL Yu J Caligiuri MA. The broad spectrum of human natural killer cell diversity. Immunity (2017) 47:820–33. 10.1016/j.immuni.2017.10.008 29166586 PMC5728700

[34] Amand M Iserentant G Poli A Sleiman M Fievez V Sanchez IP Human CD56(dim)CD16(dim) cells as an individualized natural killer cell subset. Front Immunol. (2017) 8:699 10.3389/fimmu.2017.00699 28674534 PMC5474676

[35] Lajoie L Congy-Jolivet N Bolzec A Gouilleux-Gruart V Sicard E Sung HC. ADAM17-mediated shedding of FcgammaRIIIA on human NK cells: identification of the cleavage site and relationship with activation. J Immunol. (2014) 192:741–51. 10.4049/jimmunol.1301024 24337742

[36] Amrom D Kinay D Hart Y Berkovic SF Laxer K Andermann F. Rasmussen encephalitis and comorbid autoimmune diseases: a window into disease mechanism? Neurology (2014) 83:1049–55. 10.1212/WNL.0000000000000791 25142901 PMC4166360

[37] Gough SC Simmonds MJ. The HLA region and autoimmune disease: associations and mechanisms of action. Curr Genomics (2007) 8:453–65. 19412418 10.2174/138920207783591690 PMC2647156

[38] Miyadera H Tokunaga K. Associations of human leukocyte antigens with autoimmune diseases: challenges in identifying the mechanism. J Hum Genet. (2015) 60:697–702. 10.1038/jhg.2015.100 26290149

[39] Gutierrez-Arcelus M Rich SS Raychaudhuri S Autoimmune diseases - connecting risk alleles with molecular traits of the immune system Nat Rev Genet. (2016) 17:160–74. 10.1038/nrg.2015.33 26907721 PMC4896831

[40] Glatzel A Wesch D Schiemann F Brandt E Janssen O Kabelitz D. Patterns of chemokine receptor expression on peripheral blood gamma delta T lymphocytes: strong expression of CCR5 is a selective feature of V delta 2/V gamma 9 gamma delta T cells. J Immunol. (2002) 168:4920–9. 10.4049/jimmunol.168.10.4920 11994442

[41] Pang DJ Neves JF Sumaria N Pennington DJ. Understanding the complexity of gammadelta T-cell subsets in mouse and human. Immunology (2012) 136:283–90. 10.1111/j.1365-2567.2012.03582.x 22385416 PMC3385028

[42] Groom JR Luster AD. CXCR3 ligands: redundant, collaborative and antagonistic functions. Immunol Cell Biol. (2011) 89:207–15. 10.1038/icb.2010.158 21221121 PMC3863330

[43] Kurachi M Kurachi J Suenaga F Tsukui T Abe J Ueha S. Chemokine receptor CXCR3 facilitates CD8(+) T cell differentiation into short-lived effector cells leading to memory degeneration. J Exp Med. (2011) 208:1605–20. 10.1084/jem.20102101 21788406 PMC3149224

[44] Groom JR Richmond J Murooka TT Sorensen EW Sung JH Bankert K. CXCR3 chemokine receptor-ligand interactions in the lymph node optimize CD4+ T helper 1 cell differentiation. Immunity (2012) 37:1091–103. 10.1016/j.immuni.2012.08.016 23123063 PMC3525757

[45] Vezzani A French J Bartfai T Baram TZ. The role of inflammation in epilepsy. Nat Rev Neurol. (2011) 7:31–40. 10.1038/nrneurol.2010.178 21135885 PMC3378051

[46] Vezzani A Auvin S Ravizza T Aronica E Glia-neuronal interactions in ictogenesis and epileptogenesis: role of inflammatory mediators. In: Noebels JL Avoli M Rogawski MA Olsen RW Delgado-Escueta AV, editors. Jasper's Basic Mechanisms of the Epilepsies. Bethesda, MD: Oxford University Press (2012). 22787662

[47] Devinsky O Vezzani A Najjar S De Lanerolle NC Rogawski MA. Glia and epilepsy: excitability and inflammation. Trends Neurosci. (2013) 36:174–84. 10.1016/j.tins.2012.11.008 23298414

[48] Eyo UB Murugan M Wu LJ. Microglia-neuron communication in epilepsy. Glia (2017) 65:5–18. 10.1002/glia.23006 27189853 PMC5116010

[49] Vezzani A Ravizza T Balosso S Aronica E. Glia as a source of cytokines: implications for neuronal excitability and survival. Epilepsia (2008) 49 (Suppl. 2):24–32. 10.1111/j.1528-1167.2008.01490.x 18226169

[50] Fabene PF Bramanti P Constantin G. The emerging role for chemokines in epilepsy. J Neuroimmunol. (2010) 224:22–7. 10.1016/j.jneuroim.2010.05.016 20542576

[51] Fabene PF Laudanna C Constantin G. Leukocyte trafficking mechanisms in epilepsy. Mol Immunol. (2013) 55:100–4. 10.1016/j.molimm.2012.12.009 23351392

[52] Melzer N Meuth SG Wiendl H. CD8+ T cells and neuronal damage: direct and collateral mechanisms of cytotoxicity and impaired electrical excitability. Faseb J. (2009) 23:3659–73. 10.1096/fj.09-136200 19567369

[53] Kreutzfeldt M Bergthaler A Fernandez M Bruck W Steinbach K Vorm M. Neuroprotective intervention by interferon-gamma blockade prevents CD8+ T cell-mediated dendrite and synapse loss. J Exp Med. (2013) 210:2087–103. 10.1084/jem.20122143 23999498 PMC3782053

[54] Ehling P Melzer N Budde T Meuth SG. CD8(+) T cell-mediated neuronal dysfunction and degeneration in limbic encephalitis. Front Neurol. (2015) 6:163. 10.3389/fneur.2015.00163 26236280 PMC4502349

[55] Di Liberto G Pantelyushin S Kreutzfeldt M Page N Musardo S Coras R. Neurons under T cell attack coordinate phagocyte-mediated synaptic stripping. Cell (2018) 175:458–471 e19. 10.1016/j.cell.2018.07.049 30173917

[56] Sotgiu S Murrighile MR Constantin G. Treatment of refractory epilepsy with natalizumab in a patient with multiple sclerosis. Case report. BMC Neurol. (2010) 10:84. 10.1186/1471-2377-10-84 20863362 PMC2954970

[57] Bittner S Simon OJ Gobel K Bien CG Meuth SG Wiendl H. Rasmussen encephalitis treated with natalizumab. Neurology (2013) 81:395–7. 10.1212/WNL.0b013e31829c5ceb 23794679

[58] Martin-Blondel G Brassat D Bauer J Lassmann H Liblau RS CCR5 blockade for neuroinflammatory diseases–beyond control of HIV. Nat Rev Neurol. (2016) 12:95–105. 10.1038/nrneurol.2015.248 26782333

[59] Ayzenberg I Hoepner R Kleiter I. Fingolimod for multiple sclerosis and emerging indications: appropriate patient selection, safety precautions, and special considerations. Ther Clin Risk Manag. (2016) 12:261–72. 10.2147/TCRM.S65558 26929636 PMC4767105

[60] Robert R Juglair L Lim EX Ang C Wang JH Ebert G. A fully humanized IgG-like bispecific antibody for effective dual targeting of CXCR3 and CCR6. PLoS ONE (2017) 12:e0184278. 10.1371/journal.pone.0184278 28873441 PMC5584921

[61] Liang F Giordano C Shang D Li Q Petrof BJ. The dual CCR2/CCR5 chemokine receptor antagonist Cenicriviroc reduces macrophage infiltration and disease severity in Duchenne muscular dystrophy (Dmdmdx-4Cv) mice. PLoS ONE (2018) 13:e0194421. 10.1371/journal.pone.0194421 29561896 PMC5862483

