# Supplementary material for: Evidence for Innate and Adaptive Immune Responses in a Cohort of Intractable Pediatric Epilepsy Surgery Patients
Source: Front Immunol. 2019 Jan 29;10:121. doi: 10.3389/fimmu.2019.00121 (PMC6362260; doi:10.3389/fimmu.2019.00121)
Supplement: Supplementary file 1 [file Data_Sheet_1.PDF]

Evidence for innate and adaptive immune responses in a cohort of intractable pediatric epilepsy surgery patients.

Geoffrey C. Owens*, Alejandro J. Garcia, Aaron Mochizuki, Julia W. Chang, Samuel Reyes, Noriko Salamon, Robert M. Prins, Gary W. Mathern, Aria Fallah

*Correspondence: geoffreyowens@mednet.ucla.edu

Table S1: Patient cohort

Case codes	Pathology	Gender	Affected hemisphere	Age at onset	Age at surgery
460	TSC	M	R	2 mo	3y
462	TSC	F	R	3 mo	4y
472	RE	M	L	6y	9y
475	FCD IIb	F	L	3y	5y
484	RE	M	R	9y	15y
485	HME	M	R	1 mo	8 mo
490	FCD Ic / II a	M	R	1y	14y
494	FCD Ic	F	L	3 mo	8y
495	FCD Ic	F	R	5 mo	4y
497	RE	M	L	3y	5y

TSC, Tuberous sclerosis complex; RE, Rasmussen encephalitis; FCD, Focal cortical dysplasia; HME, Hemimegalencephaly
