# Supplementary material for: Evidence for Innate and Adaptive Immune Responses in a Cohort of Intractable Pediatric Epilepsy Surgery Patients
Source: Front Immunol. 2019 Jan 29;10:121. doi: 10.3389/fimmu.2019.00121 (PMC6362260; doi:10.3389/fimmu.2019.00121)
Supplement: Supplementary file 2 [file Data_Sheet_2.PDF]

Evidence for innate and adaptive immune responses in a cohort of intractable pediatric epilepsy surgery patients.

Geoffrey C. Owens*, Alejandro J. Garcia, Aaron Mochizuki, Julia W. Chang, Samuel Reyes, Noriko Salamon, Robert M. Prins, Gary W. Mathern, Aria Fallah

*Correspondence: geoffreyowens@mednet.ucla.edu

Table S2: Median expression of immune cell markers in each cluster.

Cluster	CD45 (BILs)	CD45 (PBMCs)	CD3	CD4	CD8	TCRγδ	CD56	CD11b	CD16	CD19	CD45RA	CD45RO	CD127	CD69	HLA_DR	CD25	CD103	CXCR3	CCR4	CCR5	CCR6
**1**	3.3412	0.0021	0.0007	0.0017	0.0009	0.0001	0.0011	2.8511	0.0008	0.0009	0.0050	0.8244	0.0013	0.8356	4.1736	0.0001	0.0002	0.0013	0.0018	0.2706	0.0021
**2**	0.0008	6.5771	5.4868	4.8801	1.5760	0.0015	0.0004	0.0006	0.0006	0.0022	5.4624	0.0027	2.4048	0.6974	0.0010	0.1124	0.0007	0.0021	0.0008	0.1748	0.0008
**3**	0.0014	6.6432	0.1960	0.0023	0.6109	0.0002	4.0189	1.5767	4.9132	0.0012	5.6403	0.2208	0.0006	0.4593	0.0025	0.0430	0.0007	0.2026	0.0005	0.2504	0.0011
**4**	0.0009	6.6591	5.1650	0.4446	5.6812	0.0009	0.0012	0.0008	0.0006	0.0241	6.3233	0.0021	2.5716	0.4891	0.0008	0.0034	0.0009	2.2437	0.0006	0.4597	0.0012
**5**	0.0009	6.6389	5.3070	4.9386	1.6988	0.0013	0.0020	0.0008	0.0007	0.0030	2.0411	4.6243	1.5937	0.8071	0.0024	0.9425	0.0008	0.5715	1.5889	0.0035	0.0026
**6**	0.0010	6.4888	0.1054	0.0022	0.0251	0.0001	0.0013	0.0012	0.0007	4.7996	6.0274	0.0012	0.0012	0.1164	5.1008	0.2338	0.0009	0.0616	0.0009	0.3786	4.0872
**7**	0.0009	6.5649	0.4348	2.2147	0.1410	0.0001	0.0032	5.3272	0.0009	0.0014	2.8155	4.4974	0.0021	0.5496	3.6500	0.0173	0.0007	0.0019	0.0020	0.0009	0.0023
**8**	3.6713	0.1668	0.0011	0.0023	0.0015	0.0003	2.2803	2.4986	0.0011	0.0014	0.4448	1.2674	0.0023	0.5997	4.4938	0.0001	0.0002	0.0032	0.0040	0.4964	0.0275
**9**	0.0011	6.6222	5.5751	4.9154	1.6859	0.0019	2.3525	0.0008	0.0007	0.0026	5.5632	0.1794	2.6063	0.7830	0.0012	0.1173	0.0009	0.0041	0.0009	0.2246	0.0017
**10**	5.7364	0.0492	4.7211	0.0147	4.6434	0.0022	1.0306	0.0012	0.0011	0.0015	1.7314	3.6041	0.0010	3.5805	1.9877	0.0005	0.0008	1.0373	0.0013	2.5584	0.0008
**11**	0.0011	6.7594	5.6901	0.0034	0.1255	1.5669	0.0035	0.0441	0.0016	0.0010	4.0068	2.2343	1.7349	0.2184	0.0021	0.2089	0.0008	1.9770	0.0008	1.3814	0.0027
**12**	5.5707	0.3898	4.8157	2.5023	0.0500	0.0013	0.4839	0.0009	0.0016	0.0011	0.8390	4.8359	0.8439	3.4109	0.7408	0.0017	0.0003	0.8465	0.2682	2.5152	0.0012
**13**	5.8034	0.1641	4.6481	0.1287	5.3133	0.0007	3.2195	0.0008	0.0009	0.0020	3.2729	3.5553	0.0006	3.8371	1.4333	0.0007	3.5889	1.8909	0.0009	2.6374	0.0010
**14**	0.0011	6.7110	4.8830	0.3290	5.4006	0.0009	0.0014	0.0018	0.0010	0.0032	4.4241	1.3413	0.1015	0.6053	0.0027	0.0087	0.0009	0.6328	0.0008	0.3555	0.0014
**15**	5.6658	0.0027	4.8327	2.7957	0.0390	0.0014	0.3194	0.0004	0.0018	0.0012	0.0022	4.7607	0.0027	2.6798	2.6132	3.5487	0.0005	0.8626	2.1757	2.7795	0.0011
**16**	0.0008	7.0149	6.2829	0.1578	4.1029	2.6738	2.9094	2.0026	0.1001	0.0020	4.9927	1.5913	0.0021	0.4848	0.0020	0.3702	0.0010	2.0912	0.0002	0.5208	0.0012
**17**	0.0008	6.3704	5.0876	4.5596	1.2298	0.0009	0.0011	0.0005	0.0006	0.0017	4.5685	0.0039	0.0190	0.4805	0.0008	3.1666	0.0007	0.0018	0.0008	0.0026	0.0010
**18**	2.9597	0.3236	0.0013	0.0013	0.0011	0.0003	0.7606	1.4421	0.0016	0.0017	0.6096	0.7849	0.0031	0.1363	3.8393	0.0002	0.0006	2.5295	3.9805	0.3724	0.3352
**19**	0.0010	6.9513	5.8587	0.0031	4.1351	2.9816	0.4864	0.4835	0.4145	0.0009	5.7036	0.0372	0.0009	0.7218	0.0610	0.0676	0.0006	0.1112	0.0002	0.2816	0.0020
**20**	4.0159	2.5700	0.0025	2.3233	0.0698	0.0000	0.0035	0.0013	0.0012	0.0017	4.3017	0.0021	0.0036	0.4536	5.4520	0.0015	0.0005	2.2347	0.0166	2.2563	0.0016
**21**	0.0009	6.7534	5.2871	5.1145	1.8297	0.0013	0.0027	0.0009	0.0006	0.0165	1.7315	5.0880	3.5282	0.9169	0.0020	1.8796	0.0008	0.0060	3.6554	0.0024	3.3302

**22**	0.0007	6.6416	5.0637	4.9359	1.6678	0.0012	0.0011	0.0003	0.0003	0.0032	0.9985	4.3405	2.8997	0.7693	0.0009	2.5977	0.0005	0.0020	3.6428	0.0018	0.0018
**23**	5.5277	0.0028	5.3994	2.6930	0.0041	0.0374	0.0011	0.0008	0.0011	0.0008	4.5558	0.1508	3.0973	0.8629	0.0007	0.0002	0.0001	0.0018	0.0010	0.2760	0.0010
**24**	0.0010	5.5251	0.3609	0.0016	0.0024	0.0000	0.0039	2.9287	0.0013	0.0008	1.3423	4.4421	0.0009	0.0040	0.0021	2.7688	0.0001	0.0009	0.0007	0.4333	0.0010
**25**	0.0012	5.8918	0.6874	2.7548	0.4489	0.0001	0.0025	0.0013	0.0012	0.0011	3.7757	1.5641	0.0021	0.2472	5.5143	0.0015	0.0011	0.0379	0.0020	2.1506	0.3940
**26**	6.0480	0.3691	5.4061	0.0013	0.0015	3.0407	2.0995	0.0007	0.0009	0.0004	3.0485	4.4687	0.0008	3.8599	2.9892	0.0004	3.7080	0.0899	0.0012	2.7278	0.0005
**27**	0.0006	6.8614	5.3999	0.2085	3.7747	0.0020	1.2679	0.0011	0.0011	0.0014	2.7127	4.8282	3.9104	0.3743	0.0015	0.2354	0.0009	2.2522	0.0013	3.6387	2.9483
**28**	0.0008	7.1772	5.4024	0.5202	5.7164	0.0016	4.4091	1.7043	3.7488	0.0026	4.5516	2.5519	0.0008	0.5825	0.0043	0.1916	0.0018	1.1430	0.0008	0.4188	0.0013
**29**	0.0006	6.4066	0.0892	1.6953	0.0032	0.0003	0.0012	0.0015	4.8675	0.0011	5.2532	0.0022	0.0006	0.1098	3.4232	0.0030	0.0006	0.0020	0.0007	0.0597	0.0008
**30**	0.0006	6.6427	5.2212	4.9473	1.7277	0.0022	0.0008	0.0002	0.0011	0.0037	1.9563	3.4399	3.5748	0.8617	0.0006	2.8501	0.0005	0.0013	3.4307	0.0016	0.0017
**31**	3.0667	4.1618	0.1396	0.3255	0.0650	0.0005	1.4408	4.6466	5.3366	0.1994	3.3121	3.7199	0.8902	0.0033	0.9174	0.0175	0.0008	0.0024	0.0015	0.0020	0.7523
**32**	0.0011	6.6068	4.7988	0.2362	5.5145	0.0007	2.9352	0.0015	0.0009	0.0034	5.8350	0.0770	0.0183	0.4565	0.0015	0.0032	0.0007	0.7576	0.0007	0.3409	0.0016
**33**	4.8044	0.7100	0.0017	0.0021	0.0455	0.0002	3.6604	0.0018	1.6268	0.0013	4.5337	0.4041	0.0009	3.5723	1.8350	0.0007	3.0662	1.1639	0.0007	1.6255	0.0011
**34**	3.3418	6.5266	5.0879	4.7803	1.5279	0.0018	0.0021	2.9158	0.0008	0.0717	5.3154	1.1770	2.2955	1.5491	4.5877	0.1113	0.0013	0.1958	0.0459	0.7479	0.0039
**35**	0.0013	6.1087	0.2181	4.0663	1.1321	0.0003	0.0030	0.0008	0.0005	0.0020	6.0473	0.1043	0.0029	0.2862	5.6025	0.0014	0.0008	3.8044	0.0012	3.0051	0.0021
**36**	0.0011	6.5223	5.4301	4.8200	1.5522	0.0012	0.0014	0.0008	0.0011	0.0022	4.3193	1.8694	0.4893	0.7468	0.0010	3.4131	0.0005	0.0064	0.0024	0.0024	0.0012
**37**	0.0009	6.8334	5.3701	5.2129	2.0598	0.0015	0.1004	0.0012	0.0012	0.0029	1.6860	5.5709	4.1911	1.1774	0.0015	1.0367	0.0010	2.5535	0.0015	2.8593	3.0626
**38**	0.0010	6.6749	5.3270	4.9963	1.7130	0.0019	0.0026	0.0003	0.0006	0.0018	3.0993	3.0751	3.6449	0.8192	0.0015	2.8418	0.0012	0.0026	2.6011	0.0016	0.0020
**39**	0.0011	7.1058	0.5414	2.7733	0.4813	0.0002	0.0017	0.0014	5.8084	0.0014	6.5482	0.0039	0.0007	0.1396	4.8824	0.3805	0.0015	0.1594	0.0007	0.5215	0.0014
**40**	0.0009	6.6393	5.1190	5.0293	1.7837	0.0010	0.0020	0.0009	0.0008	0.0249	1.6220	5.0067	3.4644	0.8613	0.0011	1.0633	0.0007	0.9135	0.5959	0.0019	3.0291
**41**	5.9569	0.1323	5.0420	0.0782	5.1966	0.0012	0.2264	0.0004	0.0011	0.0018	2.2216	4.9464	0.0015	3.6862	1.9695	0.0006	2.6955	0.3661	0.0009	3.3604	0.0011
**42**	5.7289	0.0175	5.1309	0.0014	0.0015	2.0942	0.7987	0.0016	0.0014	0.0011	1.6051	3.4950	0.0014	3.6356	1.5763	0.0004	0.0004	1.0416	0.0014	2.4072	0.0008
**43**	0.0016	6.4815	5.2867	4.7663	1.4338	0.0021	2.9520	0.0010	0.0024	0.0055	4.3972	2.1990	0.8281	0.6242	0.0018	2.6486	0.0005	0.1046	0.0039	0.0278	0.0033
**44**	5.1158	0.1584	4.9769	1.3853	0.0013	0.0025	0.0933	0.0007	0.0011	0.0010	4.5493	0.1969	2.7535	1.7256	0.0018	0.0005	0.0006	0.0038	0.0017	0.1078	0.0009
**45**	0.0009	6.2920	0.3978	2.3529	0.1456	0.0000	0.0013	4.2206	0.0007	0.0011	4.2733	1.5656	0.0010	0.5049	4.0103	0.0021	0.0007	0.0017	0.0018	0.0359	0.0012
**46**	0.0005	7.0816	0.8117	2.6474	0.6447	0.0000	0.0015	4.3664	3.1645	0.0010	3.8296	3.8156	0.0028	0.4117	6.0583	0.2214	0.0033	0.0034	0.0021	0.2969	0.0023
