# Supplementary material for: Evidence for Innate and Adaptive Immune Responses in a Cohort of Intractable Pediatric Epilepsy Surgery Patients
Source: Front Immunol. 2019 Jan 29;10:121. doi: 10.3389/fimmu.2019.00121 (PMC6362260; doi:10.3389/fimmu.2019.00121)
Supplement: Supplementary file 3 [file Data_Sheet_3.PDF]

Evidence for innate and adaptive immune responses in a cohort of intractable pediatric epilepsy surgery patients

Geoffrey C. Owens*, Alejandro J. Garcia, Aaron Mochizuki, Julia W. Chang, Samuel Reyes, Noriko Salamon, Robert M. Prins, Gary W. Mathern, Aria Fallah

*Correspondence: geoffreyowens@mednet.ucla.edu

Table S3: Table of loading values for the first 19 principal components (PC).

	PC1	PC2	PC3	PC4	PC5	PC6	PC7	PC8	PC9	PC10
**cluster_1**	0.7681	0.1241	0.5807	0.03499	-0.01258	-0.007111	-0.03888	-0.002964	-0.02499	-0.01198
**cluster_2**	-0.2211	0.3557	0.06103	-0.1177	-0.1423	-0.4732	-0.1623	-0.2096	0.1257	0.0679
**cluster_3**	-0.1454	0.1234	-0.004582	0.06147	-0.193	0.6935	0.08857	-0.05209	-0.2426	-0.2589
**cluster_4**	-0.08881	0.1411	0.01342	-0.0437	-0.1808	-0.07751	0.04284	0.6056	0.464	0.06711
**cluster_5**	-0.08171	0.1234	0.02097	-0.03637	-0.1093	-0.0539	-0.004392	0.07032	0.07423	-0.319
**cluster_6**	-0.1099	0.1629	0.008063	-0.01637	-0.1624	-0.04701	-0.1149	0.02443	-0.5264	0.581
**cluster_7**	-0.1173	0.1937	0.02504	-0.09102	0.8944	0.06023	-0.05974	0.05083	0.01629	-0.03956
**cluster_8**	0.5203	0.03599	-0.7917	-0.138	-0.009811	-0.05583	-0.05738	-0.0149	-0.01533	-0.0176
**cluster_9**	-0.05282	0.08051	0.007872	-0.01969	-0.07018	0.1272	0.06806	0.3878	-0.06209	0.2142
**cluster_10**	-0.05297	-0.5048	-0.00347	0.4841	0.03774	-0.1448	-0.3664	0.1111	-0.06545	-0.06844
**cluster_11**	-0.06868	0.07958	0.009437	-0.004658	-0.03965	0.07009	0.007108	0.006659	0.08634	-0.198
**cluster_12**	-0.007471	-0.1765	-0.01712	0.2013	-0.006557	0.1732	0.2415	-0.3719	0.4403	0.3515
**cluster_13**	-0.05677	-0.5798	0.1478	-0.7608	-0.03788	0.01197	-0.04356	-0.01712	-0.02101	0.007456
**cluster_14**	-0.1053	0.1349	0.02648	-0.02734	-0.1633	-0.1678	-0.1343	-0.4254	-0.02166	-0.3163
**cluster_15**	-0.003477	-0.03664	-0.004341	0.05101	0.001628	0.004577	-0.08686	0.01374	0.004334	-0.00173
**cluster_16**	-0.001884	0.002786	0.0002127	-0.000201	-0.002984	0.001804	-0.000146	0.01093	0.002138	-0.002764
**cluster_17**	-0.01312	0.02003	0.003155	-0.004912	-0.01049	-0.01123	-0.005704	-0.05153	-0.04427	-0.03039
**cluster_18**	0.00817	0.000807	-0.009673	-0.001885	-0.000382	-0.000358	-0.000238	0.001206	-0.000178	-0.006247
**cluster_19**	-0.003472	0.004594	0.0002696	0.0001569	-0.00825	0.03682	0.01166	0.02087	-0.0362	-0.03377
**cluster_20**	-0.00145	-0.001317	-0.001324	0.006991	-0.002439	0.02312	0.005081	-0.04564	0.03589	0.03919
**cluster_21**	-0.01642	0.02492	0.00295	-0.006068	-0.02858	0.01951	0.01034	0.02855	-0.01227	-0.1199
**cluster_22**	-0.00462	0.007246	0.001308	-0.001907	-0.007499	-0.005375	-0.003219	-0.02473	-0.01181	-0.03659
**cluster_23**	-0.000236	-0.01772	-0.004728	0.02191	0.0001432	0.003122	-0.0547	0.00994	-0.001308	-0.001127
**cluster_24**	-0.00925	0.01429	0.002136	-0.006518	0.06953	0.006131	-0.002929	0.01036	0.05043	-0.04967
**cluster_25**	-0.008229	0.01279	0.0008417	-0.002877	0.01756	0.002728	-0.001278	-0.03207	-0.01351	-0.005814
**cluster_26**	-0.0257	-0.1801	0.001061	0.1752	0.05452	-0.3661	0.7635	0.04084	-0.257	-0.1186
**cluster_27**	-0.002549	0.003785	0.000549	-0.001182	-0.000124	0.003295	0.003279	0.02222	0.01525	-0.0152
**cluster_28**	-0.001176	0.001492	-0.000364	-0.000286	-0.003593	0.01588	0.005313	0.01239	-0.01202	-0.01323
**cluster_29**	-0.004398	0.006839	0.001321	-0.001616	-0.000667	-0.004608	-0.005609	-0.03717	-0.0331	0.02326
**cluster_30**	-0.002353	0.00363	0.0006349	-0.000939	-0.004195	-0.002105	-0.001091	-0.007799	-0.005655	-0.01236
**cluster_31**	0.02559	0.01199	-0.08752	-0.01923	-0.002655	0.02238	0.008852	0.04223	-0.04629	-0.0348
**cluster_32**	-0.01009	0.01501	0.0006951	-0.004241	-0.01428	0.03116	0.008131	0.02896	-0.05175	-0.007807
**cluster_33**	-0.005432	-0.03556	-0.005294	0.04866	-0.01232	0.1802	0.0629	-0.2413	0.332	0.272
**cluster_34**	0.001271	0.003333	0.00262	-0.000615	-0.00125	-0.004693	-0.001922	-0.001804	0.001889	0.0177
**cluster_35**	-0.003234	0.005867	0.0004404	-0.001872	0.0003001	0.006156	0.004345	0.02745	0.01498	-0.01385
**cluster_36**	-0.006705	0.01119	0.001038	-0.0033	-0.003926	-0.005981	0.0005208	-0.000201	0.001118	-0.01766
**cluster_37**	-0.002599	0.00401	0.0006978	-0.001729	-0.003484	-0.004761	-0.000776	0.002433	0.01032	-0.0186
**cluster_38**	-0.005353	0.008611	0.001749	-0.002563	0.00318	-0.002559	-0.000821	-0.003016	-0.004894	-0.01786
**cluster_39**	-0.003892	0.006116	0.000635	-0.001597	-0.006352	0.006692	0.004553	0.02788	0.003457	0.001312
**cluster_40**	-0.00881	0.01459	0.001559	-0.004648	-0.014	-0.01381	-0.001277	0.008472	0.02609	-0.04299
**cluster_41**	-0.003841	-0.05514	0.003019	0.01384	0.008623	-0.06171	0.1537	-0.006924	-0.03031	-0.01293
**cluster_42**	-0.03014	-0.2134	-0.003664	0.2411	0.01345	-0.03111	-0.32	0.0727	-0.02153	-0.03585
**cluster_43**	-0.004511	0.007311	-0.000347	-0.000885	-0.009721	0.02775	0.01093	0.0461	-0.0141	-0.005158
**cluster_44**	0.001256	-0.003334	-0.003627	0.004537	-0.001388	0.01416	0.000279	-0.01864	0.02332	0.01584
**cluster_45**	-0.03098	0.05383	0.009576	-0.01927	0.1577	-0.000955	-0.03191	-0.1174	-0.15	0.2273
**cluster_46**	-0.002406	0.003906	0.0004203	-0.001348	0.00759	0.001095	-0.00081	-0.001317	-0.002082	-0.000547

	PC11	PC12	PC13	PC14	PC15	PC16	PC17	PC18	PC19
**cluster_1**	-0.007415	0.03912	-0.03774	0.03893	0.00169	0.003301	0.07311	0.0307	-0.03623
**cluster_2**	-0.3645	0.3784	-0.2211	-0.06067	0.001124	0.0003467	0.2102	0.1134	-0.1107
**cluster_3**	-0.1566	0.2905	-0.1097	0.2041	-0.1682	0.001372	0.1659	0.04823	-0.06825
**cluster_4**	0.08686	-0.005564	-0.008549	0.4076	-0.1901	-0.02044	-0.07431	-0.09946	0.02151
**cluster_5**	0.3374	-0.2146	0.08235	-0.4889	-0.3533	0.02318	0.119	0.1893	-0.08282
**cluster_6**	0.3465	-0.2355	-0.1928	0.1499	-0.06631	0.01161	0.0435	0.04296	-0.006239
**cluster_7**	0.1082	-0.01295	-0.02759	0.1328	0.01661	-0.004037	0.108	0.04709	-0.07171
**cluster_8**	0.06375	0.1342	-0.03168	0.03278	0.00387	0.01284	0.07491	0.03908	-0.04602
**cluster_9**	-0.06823	0.1378	0.4484	-0.3168	0.5011	-0.02428	0.2516	0.116	-0.06961
**cluster_10**	-0.01126	0.1074	-0.03226	0.03549	0.001452	-0.2148	0.009869	0.3833	0.07254
**cluster_11**	0.1707	-0.06335	-0.5667	-0.2183	0.4967	0.01288	-0.132	-0.08107	0.09374
**cluster_12**	0.1057	-0.03694	-0.0428	-0.03926	0.01583	-0.3228	0.176	-0.1459	-0.1553
**cluster_13**	0.008762	0.05418	-0.04152	0.03494	-0.002526	0.01995	0.07525	0.01859	-0.04329
**cluster_14**	0.2275	-0.1695	0.4695	0.3829	0.2405	-0.01853	0.02355	-0.04983	0.02256
**cluster_15**	0.00996	0.02254	-0.006951	0.004769	-0.004207	0.09435	0.007419	-0.1499	0.3335
**cluster_16**	0.03257	-0.028	-0.04702	0.02701	-0.055	-0.008883	-0.05364	-0.03843	-0.05251
**cluster_17**	0.05178	-0.03209	-0.157	-0.1332	-0.001927	-0.0416	-0.1074	-0.02022	-0.03027
**cluster_18**	-0.04313	-0.0556	-0.000853	-0.001931	0.01337	-0.249	0.02688	-0.1579	0.3616
**cluster_19**	-0.04921	0.04045	-0.04261	0.1323	0.1248	0.02494	-0.1629	0.1589	-0.03858
**cluster_20**	-0.01455	0.01425	-0.009612	-0.01288	-0.07008	0.1074	-0.06719	0.0128	0.1101
**cluster_21**	0.1232	-0.09186	0.06026	-0.2118	-0.08123	-0.01746	0.04156	-0.09309	-0.08082
**cluster_22**	0.0246	-0.01727	-0.1028	-0.04165	-0.02197	-0.01459	-0.06204	0.01709	-0.001506
**cluster_23**	9.57E-05	-0.001405	-0.00649	-0.002037	-0.0106	0.1742	-0.01261	-0.043	0.2948
**cluster_24**	0.005339	-0.005511	-0.02815	0.1738	0.03089	0.05281	0.08439	0.2038	0.08844
**cluster_25**	-0.06535	0.08758	0.1113	-0.113	-0.2389	0.04681	-0.2893	-0.05617	0.1762
**cluster_26**	-0.009892	0.07208	-0.03542	0.08741	-0.009553	0.2334	0.0605	0.002119	-0.03831
**cluster_27**	0.01921	-0.01365	0.05038	-0.03061	-0.07042	0.004671	-0.00099	-0.02955	-0.03967
**cluster_28**	-0.01699	0.01727	-0.001561	0.05969	0.05451	-0.02141	-0.02318	0.009147	0.005241
**cluster_29**	-0.07571	0.0671	0.005663	-0.0228	-0.1256	-0.03301	-0.09793	0.02343	0.01141
**cluster_30**	0.02131	-0.01854	-0.0407	-0.01627	-0.01776	-0.007014	0.02783	0.008153	-0.006119
**cluster_31**	-0.6466	-0.7075	-0.03806	0.005771	0.003722	-0.0514	0.06162	0.02686	-0.04011
**cluster_32**	-0.04176	0.05068	0.09478	0.03214	0.2726	0.01196	-0.4943	-0.08843	-0.1434
**cluster_33**	0.006498	-0.08438	0.06302	-0.02507	0.01963	0.5535	-0.09032	0.3422	0.08229
**cluster_34**	-0.007319	0.008594	0.003794	0.03805	-0.08124	-0.1727	-0.3601	0.1862	-0.4715
**cluster_35**	-0.02474	0.04216	0.013	-0.03006	0.01029	0.01923	0.08385	0.06966	0.3071
**cluster_36**	0.04711	-0.03966	0.01276	0.002693	-0.01546	-0.05633	0.2039	0.0296	-0.1834
**cluster_37**	0.01645	-0.01478	0.02562	0.008127	-0.01425	-0.00487	0.01498	-0.000728	-0.02524
**cluster_38**	0.0195	-0.02298	-0.01148	0.04697	0.0469	-0.004674	0.07135	0.09389	-0.02338
**cluster_39**	0.0005244	0.001046	0.06255	0.01198	-0.07556	-0.02867	-0.01461	-0.02097	-0.08785
**cluster_40**	-0.02699	0.0418	0.04654	-0.1083	-0.002717	-0.02924	-0.2259	-0.1258	0.02431
**cluster_41**	0.01037	0.03279	-0.000586	0.03364	0.01177	-0.3273	0.05389	-0.14	0.08106
**cluster_42**	-0.04266	0.01837	-0.02373	-0.007385	0.001998	0.4539	0.1816	-0.5297	-0.3368
**cluster_43**	-0.01484	0.01633	0.07054	-0.02352	0.0573	-0.02519	-0.06316	0.06783	0.01825
**cluster_44**	0.01641	0.01999	0.0002086	0.009565	0.009835	-0.09411	0.07943	-0.2764	0.02565
**cluster_45**	-0.163	0.1764	0.2361	-0.2198	-0.202	-0.05455	-0.1819	-0.186	0.09766
**cluster_46**	-0.005239	0.001955	0.009098	0.03281	-0.05477	-0.01574	0.1957	0.05267	0.08798

PC	% variance
1	48.794
2	16.922
3	11.072
4	9.3146
5	5.6324
6	2.6612
7	2.1503
8	1.355
9	0.91184
10	0.48305
11	0.23831
12	0.17053
13	0.13754
14	0.080677
15	0.054007
16	0.011243
17	0.0069848
18	0.004465
19	0.00080194

The two variables with the highest loading values in PC1 are shown in red. The adjacent table shows the percent of variance in the data set accounted for by each PC.
