# Supplementary material for: Evidence for Innate and Adaptive Immune Responses in a Cohort of Intractable Pediatric Epilepsy Surgery Patients
Source: Front Immunol. 2019 Jan 29;10:121. doi: 10.3389/fimmu.2019.00121 (PMC6362260; doi:10.3389/fimmu.2019.00121)
Supplement: Supplementary file 4 [file Data_Sheet_4.PDF]

Evidence for innate and adaptive immune responses in a cohort of intractable pediatric epilepsy surgery patients.

Geoffrey C. Owens*, Alejandro J. Garcia, Aaron Mochizuki, Julia W. Chang, Samuel Reyes, Noriko Salamon, Robert M. Prins, Gary W. Mathern, Aria Fallah
*Correspondence: geoffreyowens@mednet.ucla.edu

Table S4: Frequency of the most abundant lymphoid and myeloid clusters.

	CD4 αβ T cell clusters						CD8 αβ T cell clusters				γδ T cell clusters			NK cell clusters		Myeloid cell clusters		
**BILs**	**2**	**5**	**9**	**12**	**15**	**23**	**4**	**10**	**13**	**14**	**11**	**26**	**42**	**3**	**8**	**1**	**7**	**45**
**460**	12.39	7.08	0.88	48.67	3.54	6.19	0	48.33	3.33	46.67	62.96	0	37.04	3.39	96.06	98.49	0.68	0.29
**462**	2.44	8.54	0	64.63	3.66	3.66	0.85	31.62	28.21	30.77	70	10	10	6.9	90.42	98.85	0.22	0.35
**472**	0.59	1.71	0	87.83	6.78	1.38	0.22	68.18	13.72	2.01	1.98	76.26	21.76	61.7	12.77	30.61	52.04	0
**475**	6.49	3.25	6.49	40.91	3.25	20.13	25.71	52.14	7.14	11.43	50	0	50	1.41	81.73	95.06	0.71	0
**484**	4.31	8.42	0	68.38	9.24	6.98	0.25	15.39	78.87	2.38	11.37	28.77	59.86	69.47	9.52	24.39	58.54	0
**485**	2.26	1.56	0.86	82.53	5.69	2.42	0.56	70.1	4.12	21.09	34.33	7.01	58.32	52.16	11.37	48.67	7.99	11.62
**490**	4.95	2.25	2.7	62.37	5.85	1.35	10.08	43.59	5.5	24.87	59.51	2.93	36.1	9.68	86.59	93.16	2.54	0.53
**494**	9.15	12.09	3.92	51.31	2.61	0.98	6.7	51.56	13.62	20.54	43.4	15.09	33.96	3.98	90.72	97.1	0.83	0.21
**495**	14.49	12.75	8.12	28.99	12.75	14.49	19.75	29.62	9.87	36.46	56.41	14.1	21.79	3.38	94.98	91.79	3.54	1.62
**497**	2.41	2.6	0	45.58	31.53	16.45	0.69	92.7	0	6.46	6.55	0.28	92.89	92.86	7.14	80.13	14.57	0
**PBMCs**																		
**460**	49.23	13.06	23.49	0.06	0	0	19.69	0.58	0	63.51	98.53	0	1.47	94.97	2.52	2.14	65.2	25.02
**462**	66.86	15.97	1.12	0.37	0	0	14.25	0.49	0.24	79.14	94.7	0	0.41	93.32	0.89	3.45	40.84	28.56
**472**	28.53	16.46	36.12	0	0.08	0	47.54	0.55	0.27	46.17	95.75	0	0.33	89.77	1.32	0.14	77.14	11.99
**475**	28.53	16.46	36.12	0	0.08	0	49.51	0.49	0.12	27.89	76.22	0	0.21	91.54	1.22	2.96	57.68	8.89
**484**	60.94	20.98	1.76	0	0	0	23.54	0.48	0.14	71.92	96.43	0	2.08	98.3	0.85	7.03	48.65	12.43
**485**	64.5	13.92	10.76	0.12	0	0.06	24.2	0.98	0.14	72.59	94.2	0	2.9	87.48	4.08	6.55	48.55	25.45
**490**	29.44	28.98	20.43	0.05	0	0	50.14	0.28	0	40.52	95.74	0	0	97	0.67	0.76	55.24	11.24
**494**	51.59	19.61	15.48	0.05	0.05	0.05	65.15	0.23	0.15	30.07	85.24	0	0	91.3	0	2.28	61.54	6.55
**495**	52.74	17.9	17.77	0.08	0.04	0	72.28	0.08	0	23.31	85.24	0	0	96.94	0.28	2.59	47.15	2.07
**497**	43.51	25.4	2.57	3.8	0.1	0.1	26.28	2	0	69.84	91.74	0	0.75	98.63	0.8	11.66	65.03	3.68

Cluster frequencies are percentages of CD4, CD8, γδ T cells, NK cells and myeloid cells respectively in each sample of BILs and PBMCs.
