# Supplementary material for: Evidence for Innate and Adaptive Immune Responses in a Cohort of Intractable Pediatric Epilepsy Surgery Patients
Source: Front Immunol. 2019 Jan 29;10:121. doi: 10.3389/fimmu.2019.00121 (PMC6362260; doi:10.3389/fimmu.2019.00121)
Supplement: Supplementary file 5 [file Data_Sheet_5.PDF]

Evidence for innate and adaptive immune responses in a cohort of intractable pediatric epilepsy surgery patients

Geoffrey C. Owens*, Alejandro J. Garcia, Aaron Mochizuki, Julia W. Chang, Samuel Reyes, Noriko Salamon, Robert M. Prins, Gary W. Mathern, Aria Fallah

*Correspondence: geoffreyowens@mednet.ucla.edu

NK cell clusters

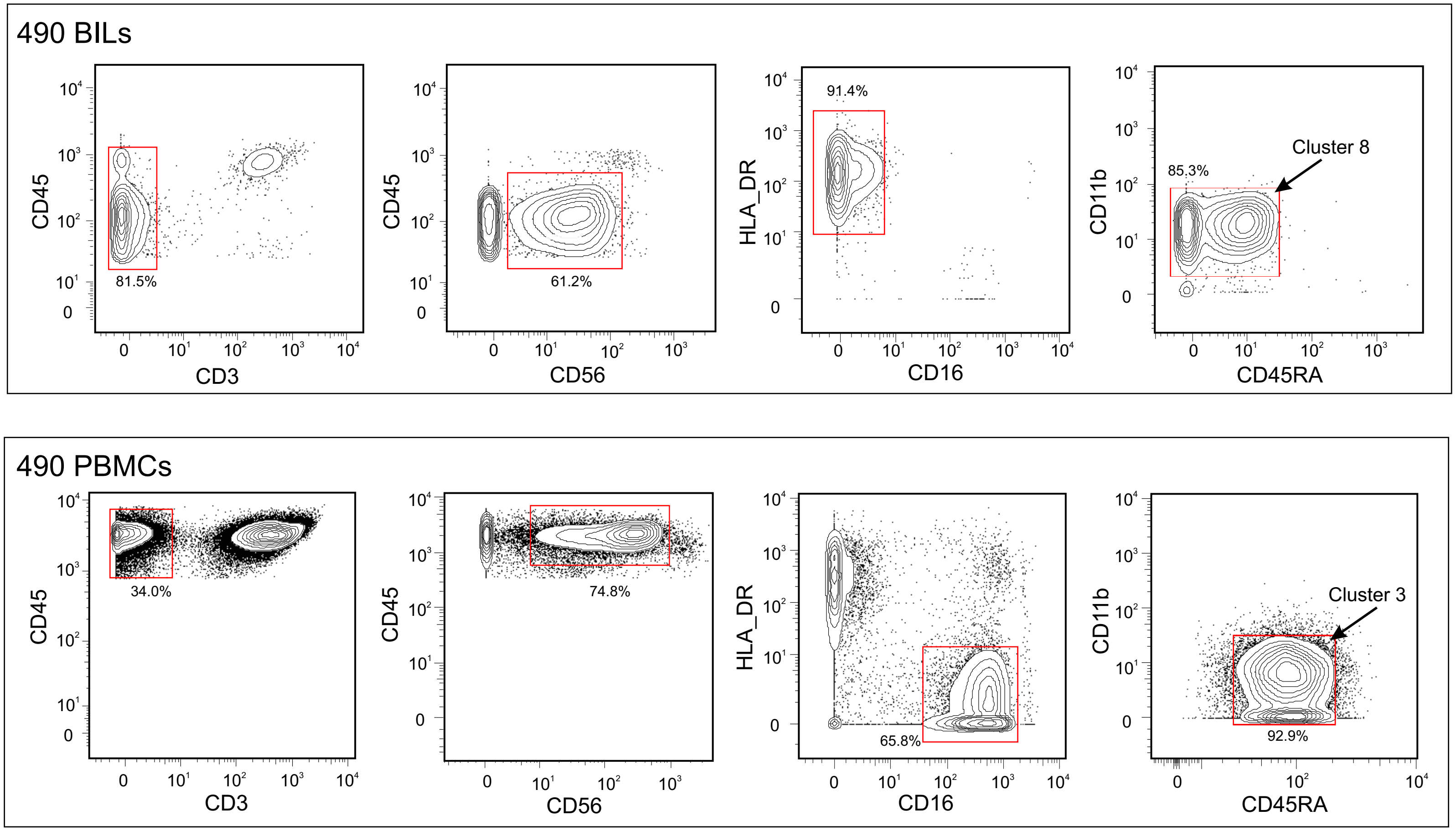

Figure S1A: Validation of Cluster 3 and 8 NK cell subsets. FCS files of CyTOF data from FCD case 490 were analyzed using FlowJoV10 software. From left to right the two dimensional contour plots (showing outliers) show the sequential gating of CD45+CD3- immune cells based on the markers that define the two NK subsets (outlined in red).

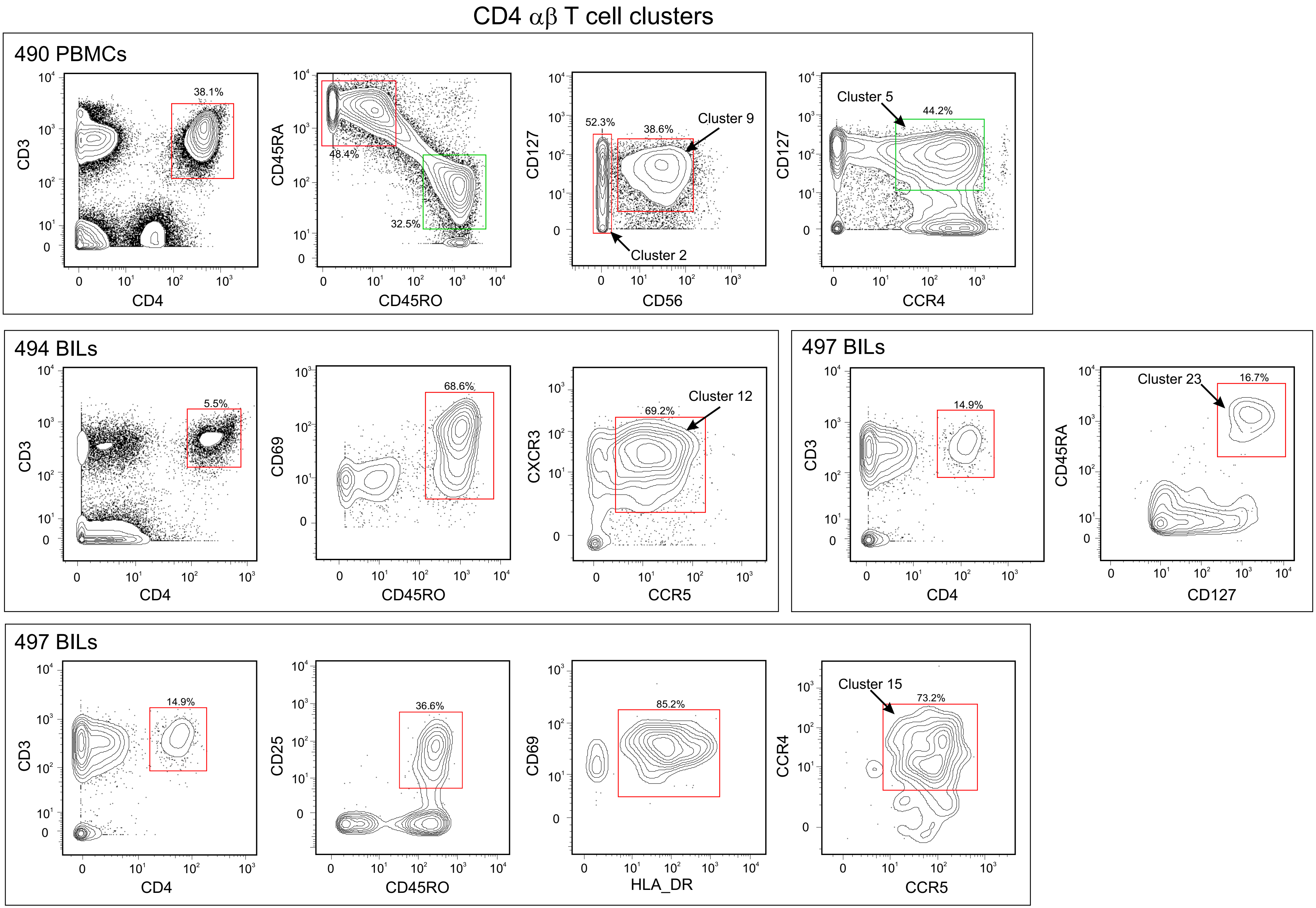

Figure S1B: Validation of CD4 T cell subsets. Two dimensional contour plots display the sequential gating of CD3+CD4+ T cells (outlined in red) by gating on the markers that defined Clusters 2, 9, 5 (top row), Clusters 12 and 23 (middle row), and Cluster 15 (bottom row). In the top row CD45RO+ cells outlined in green were subsequently gated on CD127 and CCR4

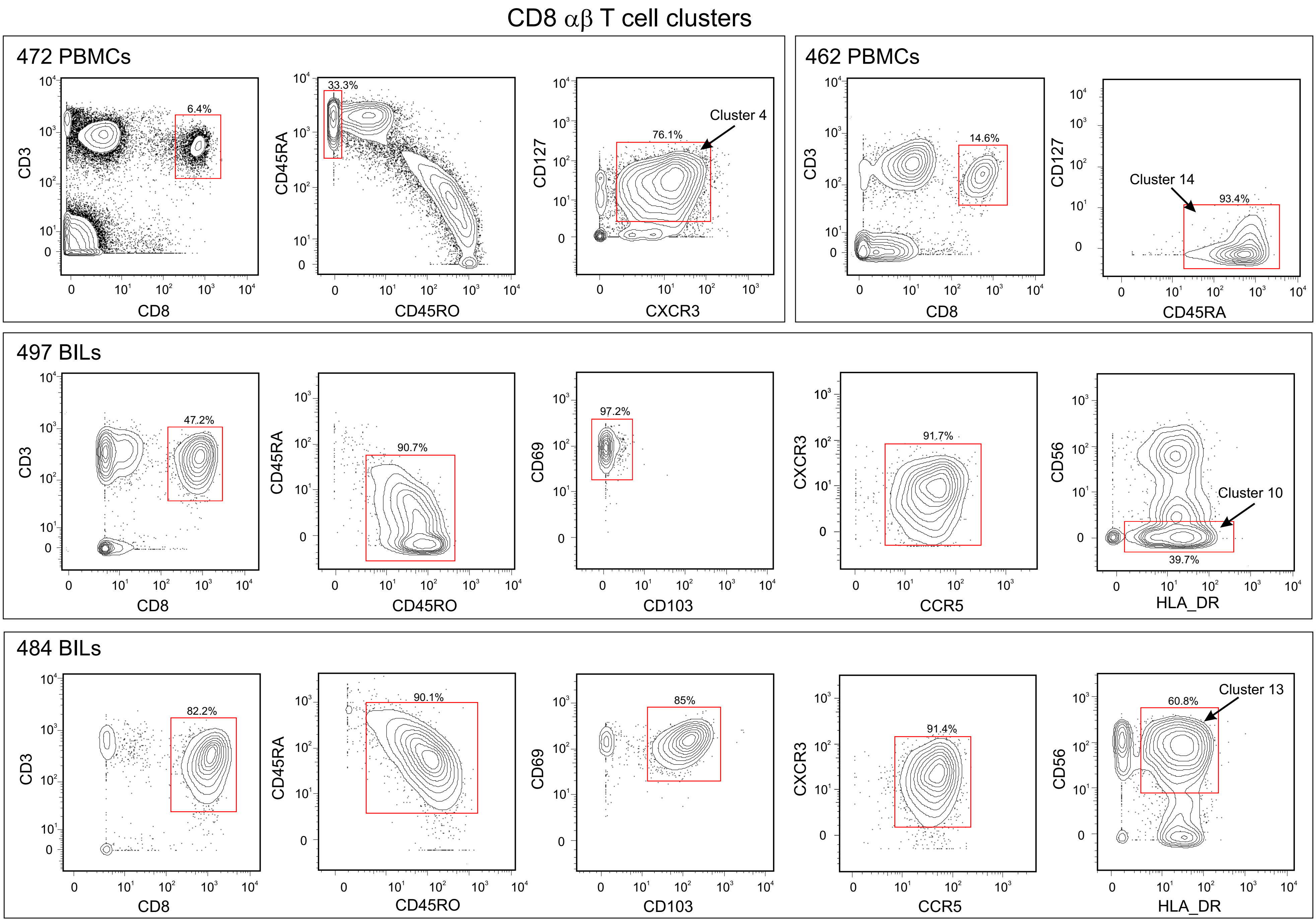

Figure S1C: Validation of CD8 T cell subsets. Two dimensional contour plots display the sequential gating of CD3+CD8+ T cells (outlined in red) by gating on the markers that defined Clusters 4 and 14 (top row), Cluster 10 (middle row), and Cluster 13 (bottom row).

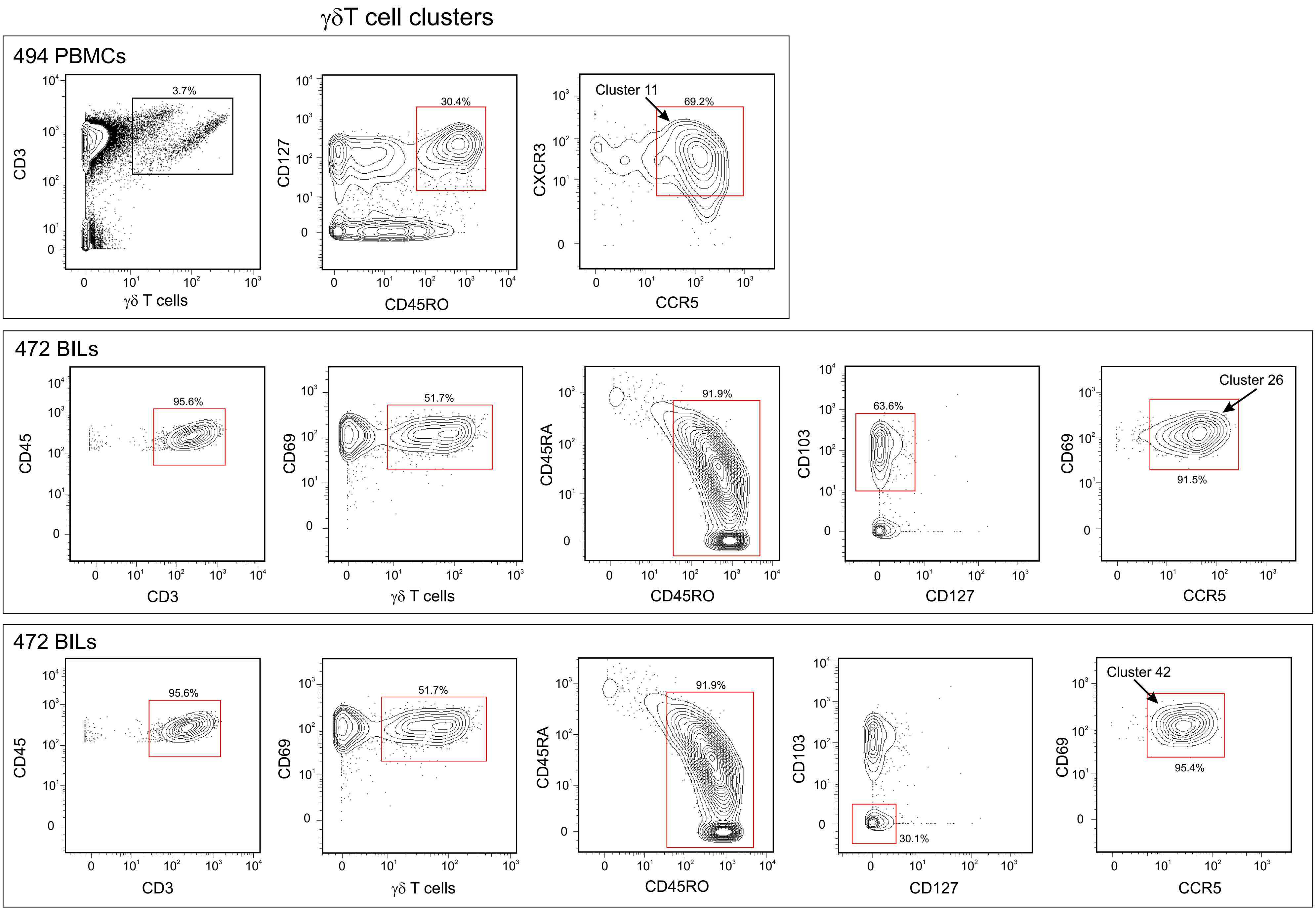

Figure S1D: Validation of γδ T cell subsets. Two dimensional contour plots display the sequential gating of CD3+γδ+ T cells (outlined in red) by gating on the markers that defined Cluster 11 (top row), Cluster 26 (middle row), and Cluster 42 (bottom row).
